# Cell-free DNA for the detection of kidney allograft rejection

**DOI:** 10.1038/s41591-024-03087-3

**Published:** 2024-06-02

**Authors:** Olivier Aubert, Cindy Ursule-Dufait, Romain Brousse, Juliette Gueguen, Maud Racapé, Marc Raynaud, Elisabet Van Loon, Angelica Pagliazzi, Edmund Huang, Stanley C. Jordan, Kenneth D. Chavin, Gaurav Gupta, Dhiren Kumar, Tarek Alhamad, Sanjiv Anand, Jorge Sanchez-Garcia, Basmah A. Abdalla, Julien Hogan, Rouba Garro, Darshana M. Dadhania, Pranjal Jain, Didier A. Mandelbrot, Maarten Naesens, Raja Dandamudi, Vikas R. Dharnidharka, Dany Anglicheau, Carmen Lefaucheur, Alexandre Loupy

**Affiliations:** 1 Université Paris Cité, INSERM U970, Paris Institute for Transplantation and Organ Regeneration, Paris, France; 2 Department of Kidney Transplantation, Necker Hospital, Assistance Publique - Hôpitaux de Paris, Paris, France; 3 Department of Nephrology and Renal Transplantation, University Hospitals Leuven, Leuven, Belgium; 4 Department of Medicine, Division of Nephrology, Comprehensive Transplant Center, Cedars Sinai Medical Center, Los Angeles, CA USA; 5 Division of Abdominal Organ Transplant, Department of Surgery, Temple University Hospital, Philadelphia, PA USA; 6 Division of Nephrology, Virginia Commonwealth University, Richmond, VA USA; 7 Division of Nephrology, Department of Internal Medicine, Washington University School of Medicine, St. Louis, MO USA; 8 Intermountain Medical Center, Transplant Services, Murray, UT USA; 9 Division of Nephrology, Department of Medicine, David Geffen School of Medicine at UCLA, Los Angeles, CA USA; 10 Department of Pediatric Nephrology, Robert Debré Hospital, Paris Cité University, Paris, France; 11 Pediatric Nephrology Department, Children Healthcare of Atlanta, Emory University, Atlanta, GA USA; 12 Weill Cornell Medicine - New York Presbyterian Hospital, New York, NY USA; 13 Department of Nephrology, Tampa General Hospital, Tampa, FL USA; 14 Department of Medicine, Division of Nephrology, University of Wisconsin School of Medicine and Public Health, Madison, WI USA; 15 Department of Pediatrics, Washington University School of Medicine, St. Louis, MO USA; 16 Kidney Transplant Department, Saint-Louis Hospital, Assistance Publique - Hôpitaux de Paris, Paris, France

**Keywords:** Diagnostic markers, Risk factors

## Abstract

Donor-derived cell-free DNA (dd-cfDNA) is an emerging noninvasive biomarker that has the potential to detect allograft injury. The capacity of dd-cfDNA to detect kidney allograft rejection and its added clinical value beyond standard of care patient monitoring is unclear. We enrolled 2,882 kidney allograft recipients from 14 transplantation centers in Europe and the United States in an observational population-based study. The primary analysis included 1,134 patients. Donor-derived cell-free DNA levels strongly correlated with allograft rejection, including antibody-mediated rejection (*P* < 0.0001), T cell-mediated rejection (*P* < 0.0001) and mixed rejection (*P* < 0.0001). In multivariable analysis, circulating dd-cfDNA was significantly associated with allograft rejection (odds ratio 2.275; 95% confidence interval (CI) 1.902–2.739; *P* < 0.0001) independently of standard of care patient monitoring parameters. The inclusion of dd-cfDNA to a standard of care prediction model showed improved discrimination (area under the curve 0.777 (95% CI 0.741–0.811) to 0.821 (95% CI 0.784–0.852); *P* = 0.0011) and calibration. These results were confirmed in the external validation cohorts (*n* = 1,748) including a cohort of African American patients (*n* = 439). Finally, dd-cfDNA showed high predictive value to detect subclinical rejection in stable patients. Our study provides insights on the potential value of assessing dd-cfDNA, in addition to standard of care monitoring, to improve the detection of allograft rejection. ClinicalTrials.gov registration: NCT05995379.

## Abstract (web summary)

A prospective observational study including 2,882 kidney allograft recipients from 14 transplantation centers in Europe and the United States shows that cell-free DNA can be used to detect kidney allograft rejection.

## Main

Allograft rejection is a major problem worldwide and results in the loss of thousands of transplants every year^1^, with devastating consequences for patients in terms of mortality, morbidity and quality of life^2^. With a prevalence of approximately 20% in the first year after kidney transplantation, allograft rejection also represents a considerable economic burden for health care systems with the associated downstream cost related to graft failure and return to dialysis^3^.

The standard strategy to monitor rejection is based on surveillance of functional markers that have remained unchanged for decades, such as serum creatinine level, estimated glomerular filtration rate (eGFR), proteinuria and circulating donor-specific anti-human leukocyte antigen antibodies (anti-HLA DSA)^4^. Although conventional biomarkers are useful in clinical practice, they lack specificity and sensitivity^5^, are subject to misinterpretation^6^, and crucially are suboptimal to detect subclinical rejection. There is therefore a critical need for improvement in the early diagnosis of rejection.

Many candidate biomarkers have been studied but few have achieved adequate prediction performance to be adopted into clinical practice as a valid patient monitoring strategy^4,7,8^. The main limitation is the quality of biomarker research lacking rigorous design, large sample size with diverse populations, robust statistical methodology, extensive external validations, accurate interpretation and transparency^9^. The current gold standard for diagnosing allograft rejection is a biopsy, which is potentially harmful, costly and often unnecessary^10^. The validation of an accurate biomarker that could reduce the need for allograft biopsy would have significant clinical and economic implications.

Cell-free DNA (cfDNA) is fragmented extracellular DNA released in the bloodstream from cells undergoing apoptosis or necrosis. Recently, it has emerged as a promising biomarker in various fields such as oncology for the detection of early stage cancer^11,12^ and for trisomy 21 screening in obstetric medicine^13^. In transplantation, dd-cfDNA detected in the blood of kidney recipients has been proposed as a noninvasive biomarker to detect rejection^14–18^. However, the association of dd-cfDNA with the presence, activity or severity of rejection has not been adequately assessed. To date, no study has evaluated the added capability of dd-cfDNA to detect allograft rejection beyond standard of care patient monitoring. As highlighted by the conclusion from the last Kidney Disease: Improving Global Outcomes (KDIGO) Conference, additional studies in different clinical scenarios and patient populations are needed to clinically validate dd-cfDNA before widespread implementation of this biomarker as a patient monitoring tool could be recommended^19^.

Therefore, the aim of this study was to assess the association of dd-cfDNA with the presence, activity and severity of allograft rejection, and to determine whether dd-cfDNA adds value to standard of care monitoring parameters in detecting kidney allograft rejection.

## Results

### Baseline characteristics of the kidney allograft recipients

**Extended Data Fig. 1:**
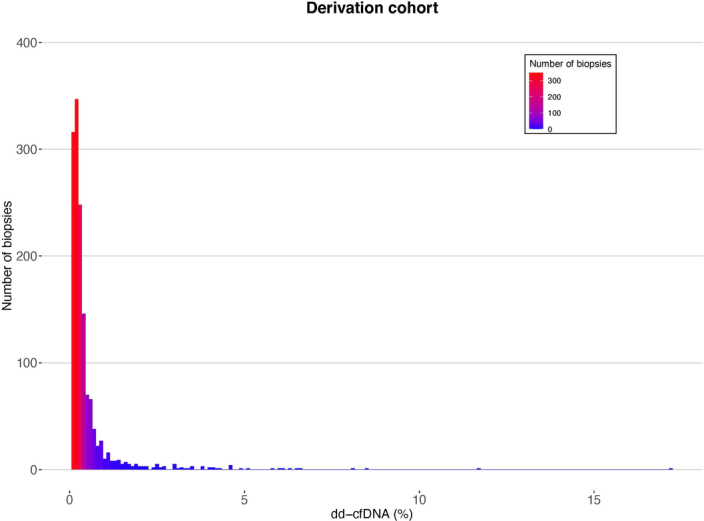
Distribution of the dd-cfDNA in the derivation cohort. This figure shows the distribution of dd-cfDNA in the derivation cohort.

All the patients with concomitant dd-cfDNA assessments and biopsies were included in this observational study to assess the association of dd-cfDNA with the presence, activity and severity of allograft rejection. The derivation cohort comprised a total of 1,134 patients corresponding to 1,415 biopsies with concomitant dd-cfDNA assessment. The mean recipient age was 55.2 ± 14.85 years, with 693 (61.11%) males. A total of 182 patients (16.05%) had a prior kidney transplant and 34 (3.02%) were ABO incompatible. The mean cold ischemia time was 14.5 ± 10.52 h. The characteristics of the recipients are summarized in Table 1. The median time between kidney transplantation and dd-cfDNA assessment was 1 year (interquartile range (IQR) 0.26–1.59) with a median percentage of dd-cfDNA of 0.27% (IQR 0.16–0.46) (Extended Data Fig. 1).

**Table 1:** Baseline patient characteristics in the derivation and validation cohort

	Derivation cohort (*n* = 1,134)		External validation cohort (*n* = 1,748)	
	*N*		*N*	
**Recipient characteristics**				
Age (years), mean (s.d.)	1,134	55.22 (14.85)	1,745	45.87 (18.14)
Sex male, *n* (%)	1,134	693 (61.11)	1,735	1,009 (58.16)
Cause of end stage renal disease, *n* (%)	1,134		1,707	
Glomerulopathy		294 (25.93)		509 (29.82)
Polycystic kidney disease		176 (15.52)		191 (11.19)
Interstitial nephritis		94 (8.29)		178 (10.43)
Diabetes		104 (9.17)		290 (16.99)
Vascular		93 (8.20)		235 (13.77)
Other		145 (12.79)		201 (11.78)
Unknown etiology		228 (20.11)		103 (6.03)
**Donor characteristics**				
Age (years), mean (s.d.)	1,129	53.77 (16.53)	1,559	42.76 (15.08)
Sex male, *n* (%)	1,127	587 (52.09)	1,240	654 (52.74)
Deceased donor, *n* (%)	1,133	820 (72.37)	1,739	1,291 (74.24)
Expanded criteria donor, *n* (%)	1,120	401 (35.80)	1,599	266 (16.64)
**Transplant baseline characteristics**				
Prior kidney transplant, *n* (%)	1,134	182 (16.05)	1,732	265 (15.30)
Cold ischemia time (hours), mean (s.d.)	1,120	14.48 (10.52)	1,260	12.92 (8.05)
HLA-A/B/DR mismatch, mean (s.d.) number	1,122	3.56 (1.52)	1,610	3.91 (1.55)
ABO incompatible transplantation, *n* (%)	1,126	34 (3.02)	1,704	19 (1.12)

### Association of dd-cfDNA with kidney allograft rejection

Among the 1,415 kidney allograft biopsies (38.2% for-cause biopsies and 61.8% protocol biopsies performed in clinically stable patients), 176 biopsies (12.44%) showed antibody-mediated rejection (AMR), including 129 (9.12%) active AMR; 34 (2.4%) revealed T cell-mediated rejection (TCMR); 17 (1.20%) were mixed rejection (AMR and TCMR); 19 (1.34%) were borderline lesions; 11 (0.78%) were inactive AMR; 20 (1.41%) were viral nephropathies; 30 (2.12%) showed glomerulitis without rejection; 48 (3.39%) were focal segmental glomerular sclerosis; 557 (39.36%) showed isolated interstitial fibrosis and tubular atrophy (IFTA); and 503 (35.55%) were biopsies with no abnormalities (none of the categories listed above). The clinical, immunological and histologic diagnoses are summarized in Supplementary Table 1 and the comparison of the patients according to the presence or absence of rejection is summarized in Supplementary Table 2.

**Fig. 1:**
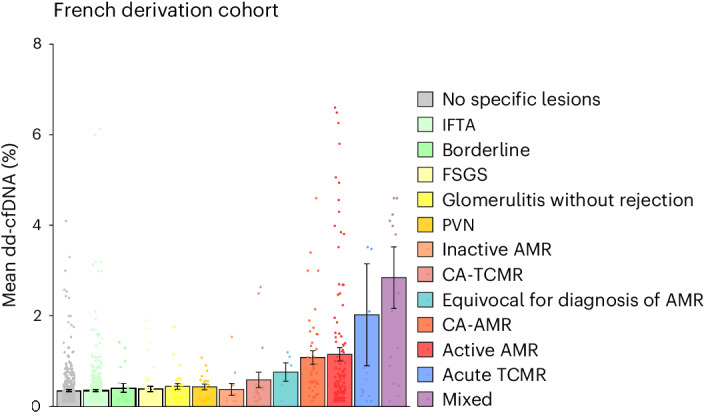
dd-cfDNA levels according to kidney allograft diagnoses. Mean level of dd-cfDNA according to the histological biopsy results. Each bar corresponds to one histological diagnosis with its mean dd-cfDNA value. Each dot corresponds to an individual dd-cfDNA value. Data are presented as mean ± s.e.m. The figure shows the increment of dd-cfDNA with active diseases (CA-TCMR, CA-AMR, active AMR, acute TCMR and mixed rejection (AMR + TCMR)). CA-TCMR, chronic active T cell-mediated rejection; CA-AMR, chronic active antibody-mediated rejection; FSGS, focal segmental glomerular sclerosis; PVN, polyomavirus-associated nephropathy. Source data

The mean dd-cfDNA was 2.85 ± 0.68% for mixed rejection, 2.03 ± 1.13% for acute TCMR, 1.15 ± 0.15% for active AMR, 1.09 ± 0.15% for chronic active AMR, 0.76 ± 0.20% for biopsies with equivocal changes of AMR, 0.59 ± 0.17% for chronic active TCMR, 0.38 ± 0.13% for inactive AMR, 0.44 ± 0.06% for polyomavirus-associated nephropathy, 0.40 ± 0.06% for biopsies with focal segmental glomerular sclerosis, 0.45 ± 0.06% for biopsies with glomerulitis without rejection, 0.42 ± 0.10% for borderline biopsies, 0.36 ± 0.02% for biopsies with isolated IFTA and 0.36 ± 0.02% for biopsies with no specific lesions (Fig. 1).

### Association of dd-cfDNA with allograft immune activity

**Fig. 2:**
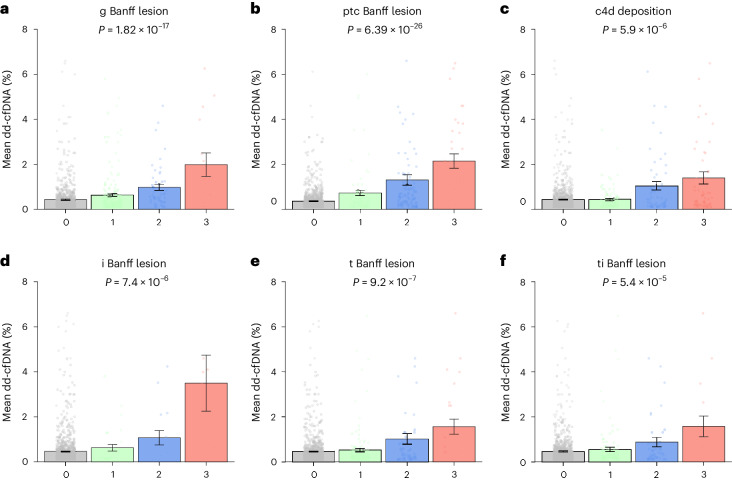
Association of dd-cfDNA with antibody-mediated lesions and TCMR lesions. **a**–**f**, Mean level of dd-cfDNA according to the Banff scores of AMR (glomerulitis (**a**), peritubular capillaritis (**b**) and c4d deposition (**c**)) and TCMR (interstitial inflammation (**d**), tubulitis (**e**) and total inflammation (**f**)). Each dot corresponds to an individual dd-cfDNA value. Each of these scores ranges from 0 to 3, with higher scores indicating more severe lesions. The lesions were defined according to Banff 2019 classification. Data are presented as mean ± s.e.m. Comparisons between the groups were performed using two-sided Kruskal–Wallis test with adjustments for multiple comparisons. This figure shows the increment of dd-cfDNA with the severity of the lesions. g, glomerulitis; i, interstitial inflammation; ptc, peritubular capillaritis; t, tubulitis; ti, total inflammation. Source data

The circulating dd-cfDNA levels were gradually increased with microcirculation inflammation severity (on a scale from 0 to 3, with higher scores indicating more severe abnormality) (Fig. 2a–c). The mean level of dd-cfDNA across lesion scores increased from 0.44 ± 0.03% to 1.99 ± 0.52% (*P* < 0.001) for glomerulitis (g Banff score), from 0.37 ± 0.01% to 2.15 ± 0.32% (*P* < 0.001) for peritubular capillaritis (ptc Banff score) and from 0.44 ± 0.02% to 1.40 ± 0.27% (*P* < 0.001) for the complement fraction c4d deposition scores.

The levels of dd-cfDNA also gradually increased with the severity of the TCMR lesions (ranging from 0 to 3) (Fig. 2d–f). The mean level of dd-cfDNA increased from 0.47 ± 0.02% to 3.50 ± 1.25% (*P* < 0.001) for interstitial inflammation (i Banff score), from 0.47 ± 0.03% to 1.57 ± 0.34% (*P* < 0.001) for tubulitis (t Banff score) and from 0.47 ± 0.03% to 1.58 ± 0.46% (*P* < 0.001) for total interstitial inflammation (ti Banff score).

Chronic allograft injuries not related to rejection including arteriosclerosis, arteriolar hyalinosis, mesangial expansion and IFTA did not show elevated dd-cfDNA (Supplementary Fig. 1).

### Independent and added value of dd-cfDNA to detect rejection

We then set out to assess whether dd-cfDNA detection was independently associated with rejection and whether it could provide additional value in addition to standard of care parameters to detect rejection. The associations of clinical, functional and immunological factors with kidney allograft rejection in univariate and multivariable logistic regression analysis are shown in Tables 2 and 3. Table 2 includes the univariate associations of each risk factor with rejection. Table 3 summarizes the multivariate logistic regression model. The model was obtained by entering the risk factors from the univariable models that met *P* ≤ 0.10 as the threshold in a single multivariable logistic regression model. Parameters included in the final multivariate model were identified using a stepwise backward elimination until each parameter was independently associated with allograft rejection with a *P* value below 0.05.

**Table 2:** Determinants of kidney allograft rejection in the derivation cohort: univariate analysis

			Number of biopsies	Number of events	OR	95% CI	*P* value
**Baseline recipient characteristics**	Age (per 1-year increment)		1,415	227	0.997	(0.987–1.006)	0.473
	Sex	Female	541	95	1	–	
		Male	874	132	0.835	(0.627–1.117)	0.221
**Baseline donor characteristics**	Age (per 1-year increment)		1,410	226	0.992	(0.983–1.000)	0.051
	Sex	Female	684	118	1	–	
		Male	724	107	0.832	(0.625–1.106)	0.206
	Donor type	Living	394	60	1	–	
		Deceased	1,020	167	1.090	(0.795–1.512)	0.599
	Expanded criteria donor	No	901	136	1	–	
		Yes	495	89	1.233	(0.918–1.650)	0.161
**Baseline transplant characteristics**	Prior kidney transplant	No	1,184	169	1	–	
		Yes	231	58	2.014	(1.427–2.814)	**5.21** **×** **10**^**−5**^
	Cold ischemia time	<12 hours	561	78	1	–	
		12–24 hours	537	89	1.230	(0.885–1.714)	
		≥24 hours	303	57	1.435	(0.984–2.083)	0.154
	Dual kidney transplant	No	1,350	212	1	–	
		Yes	65	15	1.610	(0.860–2.851)	0.117
	No. of HLA-A/B/DR mismatches		1,403	223	1.110	(1.010–1.222)	**0.032**
**Time of biopsy**	Time from transplant to biopsy (per 1-year increment)		1,415	227	1.086	(1.050–1.122)	**<2** **×** **10**^**−**^^**16**^
**Functional parameters**	eGFR (ml min^−1 ^1.73 m^−^^2^)		1,415	227	0.975	(0.967–0.983)	**5.32** **×** **10**^**−5**^
	Proteinuria (g/g) (log transformation)		1,411	227	1.434	(1.285–1.603)	**1.52** **×** **10**^**−10**^
**Clinical parameters at the time of biopsy**	Kidney graft dysfunction^a^	No	1,185	160	1	–	
		Yes	229	67	2.649	(1.898–3.674)	**7.06** **×** **10**^**−9**^
	Previous episode of rejection	No	1,316	178	1	–	
		Yes	99	49	6.265	(4.095–9.590)	**<2** **×** **10**^**−16**^
**Immunological parameters at the time of biopsy**	Anti-HLA DSA mean fluorescence intensity	<500	861	76	1	–	
		500–2,999	426	93	2.885	(2.078–4.016)	
		3,000–5,999	54	20	6.076	(3.290–10.997)	
		≥6,000	59	37	17.371	(9.833–31.383)	**<2** **×** **10**^**−16**^
	dd-cfDNA (log transformation)		1,415	227	2.599	(2.216–3.068)	**<2.2** **×** **10**^**−16**^

^a^Kidney graft dysfunction was defined according to the acute kidney injury 2012 KDIGO guidelines as an increase of serum creatinine of more than 0.3 mg dl^−1^ (>26.4 µmol l^−1^) or of more than 50% from baseline.

Association was performed using the Wald test. Data in bold are the *P* value < 0.05.

**Table 3:** Independent determinants of kidney allograft rejection in the derivation cohort: multivariable analysis

		Number of biopsies	Number of events	OR	95% CI	*P* value	OR 95% CI bootstrap BCA
**eGFR (ml** **min**^−1 ^**1.73** **m**^**−**^^**2**^**)**		1,395	226	0.989	(0.979–0.999)	**0.033**	(0.979–1.000)
**Proteinuria (g/g)** (log transformation)		1,395	226	1.180	(1.031–1.350)	**0.016**	(1.029–1.350)
**Kidney graft instability**^a^	No	1,170	159	1	–		–
	Yes	225	67	1.768	(1.144–2.711)	**0.010**	(1.102–2.894)
**Previous episode of rejection anti-HLA DSA**	No	1,300	178	1	–		–
	Yes	95	48	4.756	(2.852–7.920)	**4.754e-09**	(2.781–7.827)
**Mean fluorescence intensity**	<500	859	76	1	–		–
	500–2,999	424	93	3.081	(2.152–4.429)		(2.062–4.496)
	3,000–5,999	54	20	3.310	(1.618–6.597)		(1.726–6.607)
	≥6,000	58	37	6.338	(3.186–12.791)	**2.125e-12**	(3.410–12.258)
**dd-cfDNA** (log transformation)		1,395	226	2.275	(1.902–2.739)	**<2.2e-16**	(1.805–2.801)

The final multivariate logistic regression model was obtained by entering the risk factors from the univariable models that met *P* ≤ 0.10 as the threshold in a single multivariable logistic regression model. Parameters included in the final multivariate model were identified using a stepwise backward elimination until each parameter was associated with allograft rejection with a value below 0.05. Association was performed using the Wald test. Data in bold are the *P* value < 0.05.

^a^Kidney graft instability was defined according to the acute kidney injury 2012 KDIGO guidelines as an increase of serum creatinine of more than 0.3 mg^−^^1^ (>26.4 µmol l^−1^) or of more than 50% from baseline.

The following independent determinants of kidney allograft rejection were identified (Table 3): (1) eGFR at time of dd-cfDNA assessment (per 1 ml min^−1^ increment: odds ratio (OR) = 0.989; 95% CI 0.979–0.999; *P* = 0.033), (2) proteinuria (log transformation: OR = 1.180; 95% CI 1.031–1.350; *P* = 0.016), (3) graft instability defined as an increase of serum creatinine of more than 0.3 mg dl^−1^ (>26.4 µmol l^−1^) or of more than 50% from baseline^20^ (OR = 1.768; 95% CI 1.144–2.711; *P* = 0.010), (4) previous episode of rejection (OR = 4.756; 95% CI 2.852–7.920; *P* < 0.001), (5) anti-HLA DSAs (*P* < 0.001) and (6) dd-cfDNA (log transformation: OR = 2.275; 95% CI 1.902–2.739, *P* < 0.001). The final multivariable model was internally validated via a bootstrapping procedure with 1,000 samples from the original dataset of the derivation cohort showing consistency of the associations found in primary analyses and confirming the robustness of the final multivariable model (bias-corrected 95% CIs; Table 3).

**Fig. 3:**
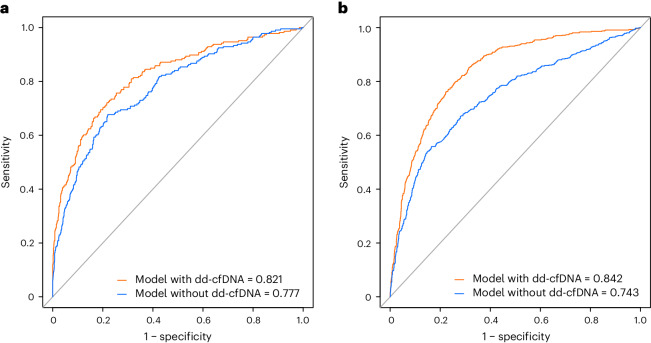
Performances of models with dd-cfDNA and without dd-cfDNA to detect kidney allograft rejection. **a**,**b**, The ROC curve, which describes the capacity of the models in discriminating rejection in the derivation cohort (**a**) and in the external validation cohort (**b**). The orange and blue lines represent the ROC curves of the models with and without dd-cfDNA, respectively. The two models also include the standard of care parameters that were independently associated with rejection in the derivation cohort (eGFR, proteinuria, anti-HLA DSA, previous episode of rejection, kidney graft instability). In those two cohorts, including dd-cfDNA beyond standard of care parameters resulted in better discrimination performances, as the AUC increased from 0.777 to 0.821 in the derivation cohort (*P* = 0.0011) and from 0.743 to 0.842 in the validation cohort (*P* < 2.2 × 10^−16^) using the Delong test. Source data

**Extended Data Fig. 2:**
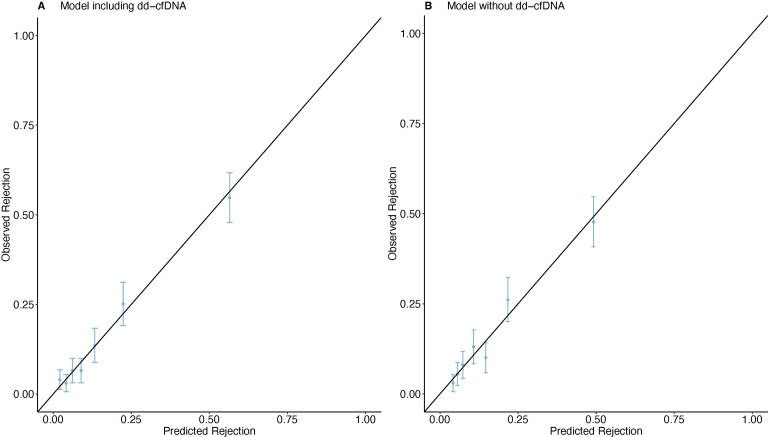
Calibration curves of the final multivariable model including dd-cfDNA versus excluding dd-cfDNA. This figure show calibration curves of the multivariable model including dd-cfDNA (panel A) and without dd-cfDNA (panel B) in derivation cohort. The x-axis represents the predicted probability of rejection and the y-axis represents the observed fraction of rejection. The bars indicates the 95% Confidence Intervals. There is a better agreement between the predictions and the observed rejection events with the model including dd-cfDNA than in the model excluding dd-cfDNA.

We finally assessed the performances of the multivariable model without dd-cfDNA and with dd-cfDNA. We showed that the inclusion of dd-cfDNA in the reference standard of care model significantly improved its discrimination capacity with an area under the curve (AUC) increasing from 0.777 (bias-corrected 95% CI 0.741–0.811) to 0.821 (bias-corrected 95% CI 0.784–0.852, *P* = 0.0011; Fig. 3a). The calibration plots were consistent with better performances of the model including dd-cfDNA compared to the model without dd-cfDNA (Extended Data Fig. 2). The added value of a dd-cfDNA over standard of care was further demonstrated by a continuous net reclassification improvement (NRI) of 0.629 for the model with the addition of dd-cfDNA compared to the standard of care model (95% CI 0.493–0.764; *P* < 0.001).

### External validation

**Extended Data Fig. 3:**
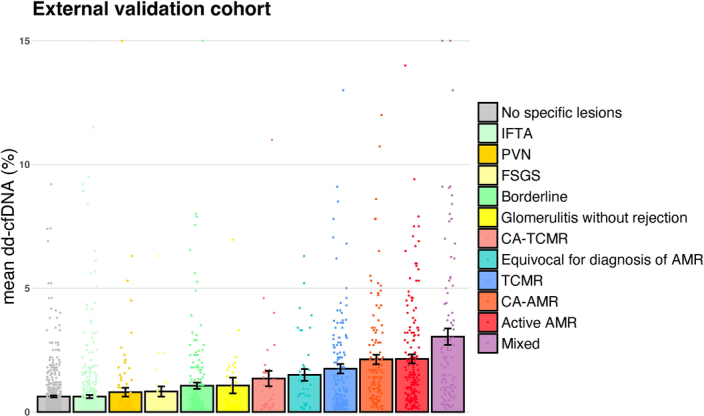
Association of dd-cfDNA according to the histologic phenotype in the validation cohort. This figure shows the mean level of dd-cfDNA according to the histological biopsy results in the validation cohort. Each bar corresponds to one histological diagnosis with its mean dd-cfDNA value. Each dot corresponds to an individual dd-cfDNA value. This figure shows the increment of dd-cfDNA with active diseases (Chronic-active-T-cell mediated rejection, Chronic-Active antibody-mediated rejection, Active antibody mediated rejection, Acute T-cell mediated rejection and mixed rejection (AMR + TCMR)). Data are presented as mean values +/- SEM.

**Extended Data Fig. 4:**
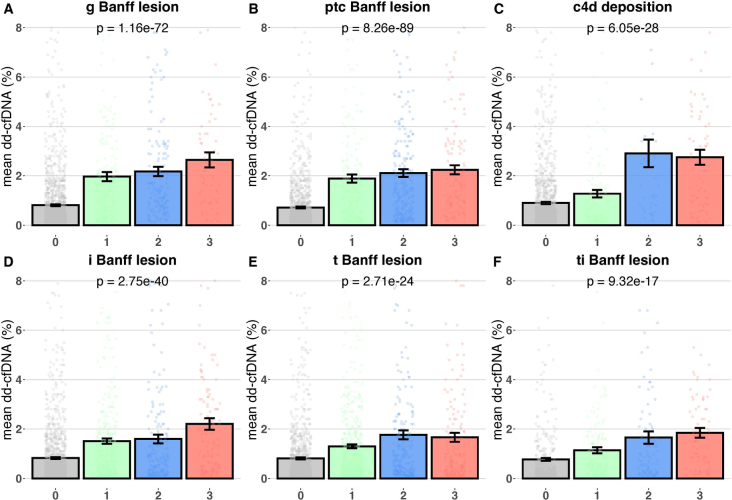
Association of dd-cfDNA with Banff scores in the validation cohort. This figure shows the mean levels of dd-cfDNA according to the ABMR features (g Banff score [glomerulitis], ptc Banff score [peritubular capillaritis], c4d graft deposition) and the TCMR features (i Banff score [interstitial inflammation], t Banff score [tubulitis], and ti Banff score [total inflammation]). Each of these scores ranges from 0 to 3, with higher scores indicating more severe lesions. The T bars indicates standard errors. Each dot corresponds to an individual dd-cfDNA value. Data are presented as mean values +/- SEM. Comparisons between the groups was performed using two-sided Kruskal-Wallis test with adjustments for multiple comparisons. This figure shows the increment of dd-cfDNA with the severity of the lesions.

The validation cohort comprised 1,748 patients and 2,317 evaluations from 12 transplantation centers. The characteristics of the recipients are summarized in Table 1. The median time between kidney transplantation and dd-cfDNA assessment was 0.85 (IQR 0.26–2.05) years. The median dd-cfDNA was 0.40% (IQR 0.19–1.20) in the external validation cohort (Supplementary Fig. 2). The different biopsy findings are detailed in Supplementary Table 3 and the comparison of the patients according to the presence or absence of rejection is summarized in Supplementary Table 4. Overall, 352 (15.19%) biopsies showed AMR with 221 (9.54%) active AMR, 224 (9.67%) showed TCMR and 103 (4.45%) mixed rejections. Extended Data Fig. 3 shows the association of dd-cfDNA according to the diagnoses in the validation cohort. The increased values of dd-cfDNA with the severity of Banff active lesions were confirmed in the validation cohort (Extended Data Fig. 4).

In multivariable analyses, dd-cfDNA (log transformation) remained associated with rejection in the validation cohort (OR = 2.317, 95% CI 2.083–2.588; *P* < 0.001), independently of standard of care factors (eGFR, proteinuria, kidney graft instability, previous episodes of rejection and anti-HLA DSA) (Extended Data Table 1). We also showed that the inclusion of dd-cfDNA in the reference model significantly improved the discrimination capacity in the validation cohort as the AUC increased from 0.743 (bias-corrected 95% CI 0.717–0.766) to 0.842 (bias-corrected 95% CI 0.820–0.858) (*P* < 0.001) (Fig. 3b). The added value of dd-cfDNA over standard of care was further demonstrated by a continuous NRI of 0.853 (95% CI 0.764–0.943; *P* < 0.001) for the model with the addition of dd-cfDNA compared to the standard of care model.

### Generation of an integrative dd-cfDNA score to detect rejection

**Extended Data Fig. 5:**
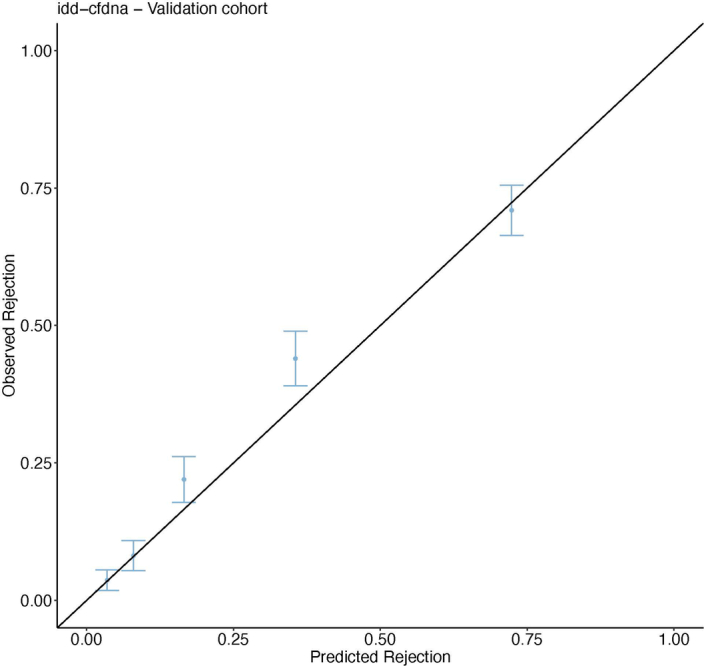
Calibration curve of the integrative dd-cfDNA score in the validation cohort. This figure shows the calibration curves of the integrative dd-cfDNA score in the validation cohort. The x-axis represents the predicted probability of rejection and the y-axis represents the observed fraction of rejection. The bars indicates the 95% Confidence Intervals.

We then aimed to generate an integrative score including dd-cfDNA and the standard of care parameters to detect rejection and assess its performance. A multidimensional rejection risk score integrating dd-cfDNA and standard of care parameters, the integrative dd-cfDNA (idd-cfDNA), was calculated for each patient according to the β-regression coefficients estimated from the final multivariable model. The score demonstrated a good discrimination with an AUC of 0.821 (bias-corrected 95% CI 0.784–0.852) and a precision–recall area under the curve (PRAUC) of 0.576 (bias-corrected 95% CI 0.509–0.634) (Fig. 3a). Brier score for idd-cfDNA was at 0.099 in the derivation cohort, which was also consistent with a good calibration (Extended Data Fig. 2a). The score demonstrated good performance in the external validation cohort with an AUC of 0.838 (bias-corrected 95% CI 0.817–0.855) (Supplementary Fig. 3), a PRAUC equal to 0.671 (bias-corrected 95% CI 0.626–0.707) and a good calibration (Extended Data Fig. 5). The AUC was equal to 0.815 (bias-corrected 95% CI 0.735–0.869) and 0.829 (bias-corrected 95% CI 0.807–0.850) in Belgium and North America respectively. In the pediatric kidney transplant recipients (*n* = 197 patients with 338 evaluations), the AUC was equal to 0.800 (bias-corrected 95% CI 0.711–0.862). The performance metrics of this integrative score are given in Supplementary Table 5.

### Sensitivity analyses

Various sensitivity analyses were performed to test the association and added value of dd-cfDNA in different clinical scenarios and subpopulations. These included:

1. Timing of dd-cfDNA measurement. We found that the independent association of dd-cfDNA with allograft rejection remained significant when dd-cfDNA was assessed in the first 3 months posttransplantation (OR = 2.370; 95% CI 1.505–3.730, *P* < 0.001), between 3 months and 1 year posttransplantation (OR = 1.882; 95% CI 1.381–2.566, *P* < 0.001) or after 1 year posttransplantation (OR = 2.521; 95% CI 1.944–3.316, *P* < 0.001) (Extended Data Table 2).
2. Rejection phenotypes. We also confirmed the robustness and the incremental value of dd-cfDNA over standard of care (including anti-HLA DSA) to detect AMR (AUC = 0.840; 95% CI 0.807–0.870) and TCMR (AUC = 0.780; 95% CI 0.683–0.845) (Extended Data Tables 2 and 3).
3. Added value of dd-cfDNA to detect allograft rejection in stable patients (with stable kidney function (eGFR) and without proteinuria). We found that dd-cfDNA was also independently associated with subclinical rejection (OR = 2.200; 95% CI 1.663–2.947, *P* < 0.001), showing a 66.3% reclassification of cases with subclinical rejection (subclinical AMR and subclinical TCMR in patients with stable kidney function and no proteinuria) over standard of care (NRI 0.6254; 95% CI 0.408–0.843; *P* < 0.001) (Extended Data Tables 2 and 3).
4. In African American patients in the United States external validation cohort. We found that dd-cfDNA was also independently associated with rejection in African American recipients (OR = 2.816; 95% CI 2.216–3.349, *P* < 0.001) and confirmed the robustness and the incremental value of dd-cfDNA over standard of care to detect rejection (AUC = 0.833; 95% CI 0.794–0.870) in this subpopulation.

Association of dd-cfDNA with the movement of diagnosis and response to treatment was assessed. In total, 639 patients with at least two assessments of dd-cfDNA during their follow-up were included. The median time between the two dd-cfDNA assessments was 6.51 (IQR 2.92–11.73) months. Four distinct immunological statuses were identified: (1) patients with immune quiescence (normal biopsies over time) (*N* = 386), (2) patients with de novo allograft rejection (a normal biopsy followed by subsequent allograft rejection occurring at a later time point) (*N* = 89), (3) patients with treated allograft rejection (successfully treated rejection episode with normal biopsy after rejection treatment) (*N* = 75) and (4) patients with persisting allograft rejection (persistence of immune activity after treatment) (*N* = 89). The movements of dd-cfDNA were associated with the clinical scenarios showing a stability for the patient with immune quiescence (*P* = 0.3472), a significant increase for patients with de novo allograft rejection (*P* < 0.0001), a significant decrease for patients with treated rejection (*P* < 0.0001) and a significant decrease for patients with persisting allograft rejection after treatment (*P* = 0.0020), but these patients still display persistent elevated dd-cfDNA (mean dd-cfDNA: 1.33 ± 0.17%) (Extended Data Table 4).

Decision curve analysis was performed to assess the clinical value of the model integrating dd-cfDNA. In comparison, decision-making using the model integrating dd-cfDNA provided greater net clinical benefit to patients for all decision thresholds compared to standard of care clinical assessment (Supplementary Fig. 4).

**Extended Data Fig. 6:**
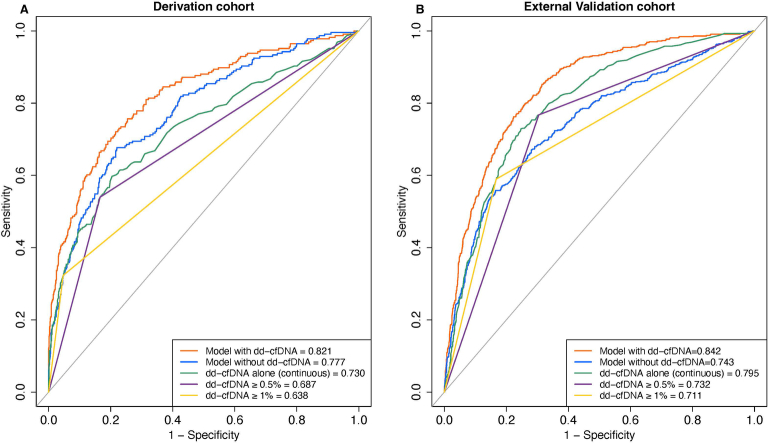
ROC curves of the different models and of dd-cfDNA as a continuous biomarker or with specific thresholds (0.5 or 1%) in the derivation and validation cohorts. Panel A shows the receiver-operating (ROC) curve of different models (model including or without dd-cfDNA), and of dd-cfDNA alone, with or without thresholds. Panel B depicts the ROC curves in the validation cohorts.

We also conducted a comparison of the performance metrics, including sensitivity, specificity, negative predictive value, positive predictive value, AUC, PRAUC and Brier score of dd-cfDNA when used as a single biomarker to detect rejection. We employed different approaches to interpret dd-cfDNA results, such as using it as a continuous parameter or applying specific thresholds based on existing literature (0.5% and 1%). In both derivation (Supplementary Table 6) and validation cohorts (Supplementary Table 7), using dd-cfDNA as a continuous parameter improved all the performance metrics (except specificity and positive predictive value) in contrast to the use of fixed thresholds. However, the best performance metrics were reached when dd-cfDNA was used in combination with standard of care parameters, highlighting the necessity to combine it into an integrative score. The receiver-operating characteristic (ROC) curves of the different models in the derivation and validation cohorts are depicted in Extended Data Fig. 6a,b.

## Discussion

In this large multinational study, we demonstrated that elevated levels of dd-cfDNA were highly associated with the presence, activity and severity of all types of kidney allograft rejection, and showed its added value beyond standard of care monitoring in predicting rejection. We demonstrated the generalizability of the association of dd-cfDNA with rejection and its added value by showing its external validity in 12 geographically distinct and multi-ethnic cohorts recruited in Europe and the United States with distinct allocation systems, patient characteristics and management practices.

We found that dd-cfDNA was not only associated with rejection but that higher levels of dd-cfDNA were associated with a higher degree of activity and severity of AMR or TCMR. We also demonstrated the incremental increase of dd-cfDNA with the severity of microcirculatory inflammation, complement fraction c4d deposition, tubulitis, interstitial inflammation and total inflammation. We demonstrated that dd-cfDNA was able to detect any kind of rejection, including subclinical rejection, which remains the most challenging diagnosis when using traditional kidney transplant markers alone. Contrary to previous studies, which have reported that recipients with elevated dd-cfDNA but preserved allograft function remain stable over follow-up^21^, we showed the independent association and the added value of dd-cfDNA to detect subclinical rejection in recipients with stable allograft function.

In this study, dd-cfDNA was not associated with polyomavirus-associated nephropathy or recurrence of glomerulonephritis. However, because rejection is higher after minimization in case of viral infection, dd-cfDNA might help to monitor the patients better and detect earlier rejection following treatment decrease or withdrawal^22^.

In our cohort, active AMR was found in 9.12% (*n* = 129 of 1,415) of patients in the derivation cohort and 9.54% (*n* = 221 of 2,317) of patients in the external validation cohort. This incidence aligns well with the most recently reported incidence of AMR ranging from 3% to 12% in a recent systematic review including 28 studies^23^. Higher levels of dd-cfDNA in cases of TCMR compared to AMR was observed within the derivation cohort, in contrast to the validation cohort where elevated dd-cfDNA levels were more pronounced for AMR compared to TCMR. However, no statistically significant differences in dd-cfDNA levels were found between AMR and TCMR in either cohort. Notably, there were no significant differences in dd-cfDNA levels between the two cohorts for TCMR. Only AMR demonstrated significantly higher levels of dd-cfDNA levels in the validation cohort when compared to the derivation cohort. This discrepancy can be attributed to the severity of the biopsies in the validation cohort, which were mostly for-cause biopsies with more severe AMR cases, as shown by the Banff g score.

Long-held conventional wisdom suggested the best way to monitor allograft function and risk for rejection was systematic analysis of serum creatinine^4^. In our study dd-cfDNA was challenged by not only comparing dd-cfDNA to functional variables such as creatinine but also to all the markers currently used in routine posttransplantation clinical practice, including recipient and donor characteristics, serum creatinine/eGFR, proteinuria and anti-HLA DSA. We demonstrated that while the conventional markers widely used in kidney transplant are associated with a good performance (AUC 0.777) to detect rejection, the addition of dd-cfDNA not only significantly increased the discriminative capability in our French cohort (AUC 0.821, *P* = 0.001) but also in cohorts from the United States and Europe, with the AUC increasing from 0.748 to 0.845 with the addition of dd-cfDNA (*P* < 0.001).

Most new biomarkers have been developed specifically for AMR, and most studies do not make a distinction between TCMR and AMR. Liquid biopsies involving circulating cfDNA have rapidly emerged as an important and minimally invasive adjunct to standard biopsies and, in some cases, even a potential alternative approach for rejection detection and monitoring. We showed that dd-cfDNA was independently associated with AMR and TCMR when measured at different timepoints posttransplantation, reinforcing its utility in clinical practice.

Moreover, this study shows the independent association of dd-cfDNA with AMR independently of anti-HLA DSA. dd-cfDNA detects AMR and TCMR separately and adds value to DSA to better stratify patients at risk of rejection. The inclusion of dd-cfDNA to a standard of care prediction model showed improved discrimination, calibration and significant rejection risk reclassification compared with standard of care patient monitoring including anti-HLA DSA (*P* < 0.001).

This study opens avenues to improve the management of renal transplant patients by more specifically tailoring the clinical indication of biopsies. While the diagnosis of rejection still relies on biopsies, the addition of dd-cfDNA in clinical practice to the standard of care monitoring factors may improve the early diagnosis of rejection and therefore optimize the safety, management and care of kidney allograft recipients.

Regarding the study limitations, we acknowledge that the assessment of the value of repeated dd-cfDNA measurements as a longitudinal biomarker of the occurrence, recovery or progression of rejection episodes was only performed in a subset of patients. Longitudinal assessment of dd-cfDNA has the potential to be useful in this context, as it has been shown that elevation of dd-cfDNA occurs ahead of clinically apparent organ injury in heart, lung and kidney transplant recipients^24–26^.

Second, information regarding drug adherence, which is a factor in rejection, was lacking in our dataset. Although nonadherence is inherently difficult to capture, especially at a population level^1^, dd-cfDNA has the potential to capture the consequences of nonadherence early before the appearance of histological lesions.

Third, the present study populations show great similarity compared with US, European and French registries, thereby reinforcing the generalizability of the results, which were validated in different centers from Europe and in the United States, and in various clinical scenarios including pediatric patients. In our study, 30.8% (*n* = 439 of 1,426) of the US validation cohort comprises African American patients. Independent analysis of the model, although trained on data from patients of different ethnicities, showed a good performance in this critically underserved population with worse outcomes after transplantation. Validation of the score in a large number of patients from other ethnicities is needed to assess the generalizability of the conclusions.

Lastly, although the association of dd-cfDNA with rejection and its added value beyond standard of care factors were assessed in a large, unselected cohort, future clinical trials are needed to investigate whether a strategy using dd-cfDNA to detect rejection is clinically relevant.

We have demonstrated that levels of circulating dd-cfDNA are strongly associated with the presence, activity and severity of kidney allograft rejection and that assessment of circulating dd-cfDNA improves the detection of allograft rejection beyond standard of care patient monitoring.

## Methods

### Inclusion and ethics statement

Our research complies with all relevant ethical regulations. The protocol of this study (NCT05995379) was approved by the Paris Transplant Group’s Institutional Review Board (IRB). All data were anonymized, and the clinical and biological data were collected from each center and entered into the Paris Transplant Institute database (French data protection authority (CNIL) registration number 363505). The protocol of this study (NCT05995379) was approved by the Paris Transplant Group’s IRB. Data were retrieved from the database in November 2022. The database networks have been approved by the National French Commission for bioinformatics data and patient liberty and codes were used to ensure strict donor and recipient anonymity and blind access. Informed consent was obtained from the participants at the time of transplantation and from parents or carers for pediatric patients. All data were anonymized and prospectively entered at the time of transplantation, and were updated at several timepoints (3, 6 and 12 months posttransplantation and then annually) and at each clinical event using a standardized protocol to ensure harmonization across study centers. Data from the derivation cohort were submitted for an annual audit to ensure data quality. As part of standard clinical procedures, the external validation datasets from the European and North American centers were compiled, entered in the databases of the centers in accordance with local and national regulatory standards, and submitted to the Paris Transplant Institute anonymously. There was no participant compensation.

### Study population

In this multinational population-based study, 2,882 patients from 14 centers were included. The derivation cohort consisted of 1,134 patients over 18 years of age prospectively recruited in two French centers (Necker Hospital, Paris and Saint-Louis Hospital, Paris) between 17 April 2013 and 21 June 2021. Patients with combined organ transplantation, pregnant women, recipients of a graft from a monozygotic twin and patients who had received a bone marrow transplant were excluded. Data were retrieved from the database in November 2022. Both sexes were included and self-reported. Research findings applied to both sexes and data were reported disaggregated for sex.

External validation was conducted on 1,748 adult and pediatric patients from 12 centers including 1,426 patients in 11 North American centers and 322 patients in one Belgian center. In the US external validation cohort, self-reported information on ethnicity was collected from records. In the US validation cohort, 39.27% were white, 30.79% were African American, 5.96% were Asian, 0.35% were Hawaiian, 10.45% were Hispanic, 3.16% were of other ethnicities and 10.03% did not report their ethnicity. Ethnicity was not recorded in the other cohorts. External validation was conducted on 1,748 kidney transplanted patients from 12 transplantation centers including 322 patients in one Belgium center (Leuven Hospital) and 1,426 patients in eleven North American centers (Cedars-Sinai, Los Angeles, CA (*N* = 284), UCLA, Los Angeles, CA (*N* = 72), University Hospitals, Cleveland, OH (*N* = 30), Emory University, Atlanta, GA (*N* = 136), Intermountain Medical Center Transplant Services, Murray, UT (*N* = 51), Weill Cornell Medical School and New York Hospital Medical Center, New York, NY (*N* = 34), Virginia Commonwealth University, Richmond, VA (*N* = 327), Pediatrics Center and Adults Center, Washington University School of Medicine in St. Louis, St. Louis, MO (*N* = 153), Transplant Institute, Tampa General Hospital, Tampa, FL (*N* = 132) and University of Wisconsin–Madison, Madison, WI (*N* = 207).

The Belgian and North American validation cohorts followed the rules applied in each country. In these centers, datasets were collected as part of routine clinical practice and entered in centers’ databases in compliance with local and national regulatory requirements, and sent anonymized to the Paris Transplant Group.

### Detection of donor-specific anti-HLA antibodies

All patients were tested for the presence of circulating anti-HLA DSAs at the time of patient risk evaluation^27^. The presence of circulating DSAs against HLA-A, HLA-B, HLA-Cw, HLA-DR, HLA-DQ and HLA-DP was retrospectively determined using single-antigen flow bead assays (One Lambda, Inc.) on a Luminex platform. Beads with a normalized mean fluorescence intensity, a measure of DSA strength, of greater than 500 units were judged as positive as previously described. HLA typing of the transplant recipients and donors was performed using an Innolipa HLA Typing Kit (Innogenetics). In the validation cohorts, HLA genotyping and HLA antibody profiling were performed according to local center practice

### Kidney allograft phenotypes at the time of risk assessment

In the derivation cohort, allograft biopsies were scored and graded from 0 to 3 according to the updated Banff criteria for allograft pathology for the following histological factors^28^: glomerular inflammation (glomerulitis), tubular inflammation (tubulitis), interstitial inflammation, endarteritis, peritubular capillary inflammation (capillaritis), transplant glomerulopathy, interstitial fibrosis, tubular atrophy, arteriolar hyalinosis and arteriosclerosis. Additional diagnoses provided by the biopsy (for example, the diagnoses of primary disease recurrence, BK virus nephropathy) were recorded. The biopsy sections (4 μm) were stained with periodic acid-Schiff, Masson’s trichrome, and hematoxylin and eosin. C4d staining was performed via immunohistochemical analysis on paraffin sections using polyclonal human anti-C4d antibodies. Also in the validation cohorts, the Banff criteria for the individual histological lesions were assessed in each biopsy included in the study.

### Assessment and measurement of donor-derived cfDNA

Plasma samples were collected in heparin coated tubes or Cell-Free DNA BCT tubes (Streck) at the time of allograft biopsy performed for clinical indication or as per protocol, which was performed after transplantation according to the centers’ practices. Plasma samples were shipped to CareDx, Inc. for dd-cfDNA analysis. Extracted cfDNA was assessed using the targeted next-generation sequencing assays (AlloSure or AlloSeq cfDNA, CareDx) and dd-cfDNA was quantified as a percentage of total cfDNA^29^. All cfDNA analyses were performed blindly in the absence of any clinical, biological and/or histological data.

### Clinical data

Clinical data comprised demographic parameters including recipient’s age, sex and transplant characteristics; biological parameters, including kidney allograft function, proteinuria, and anti-HLA DSA specificities and levels; and allograft pathology data, including Banff lesion scores and diagnoses.

Kidney allograft function was assessed by the glomerular filtration rate estimated by the Modification of Diet in Renal Disease Study equation (eGFR) and proteinuria level using the protein/creatinine ratio. Graft instability was defined according to the acute kidney injury 2012 KDIGO guidelines as an increase of serum creatinine of more than 0.3 mg dl^−1^ (>26.4 µmol l^−1^) or of more than 50% from baseline. Baseline serum creatinine was considered as the lowest serum creatinine value during the month before biopsy or the last known serum creatinine.

### Outcome measure

The outcome of interest was biopsy-proven rejection comprising AMR, TCMR and mixed rejection. Allograft rejection was detected in screening allograft biopsies performed at 3 months and 1 year after transplant and/or in for-cause biopsies performed at any time posttransplantation in unstable patients. Pathologists and physicians were blinded to dd-cfDNA results.

### Statistical analysis

Continuous variables were described by using means and standard errors or standard deviations or medians and interquartile ranges. We compared means and proportions between groups by using Student’s *t*-test, analysis of variance (or Mann–Whitney test if appropriate) or the chi-squared test (or Fisher’s exact test if appropriate).

We evaluated the association between the dd-cfDNA levels and each allograft diagnosis (AMR, TCMR, mixed rejection, glomerulitis without rejection, polyomavirus nephritis, fibrosis, borderline changes, no specific lesions). To investigate the association of dd-cfDNA with rejection severity, we compared the mean dd-cfDNA level according to each active and chronic Banff lesion score (ranked from 0 to 3).

The associations between allograft rejection and clinical, functional immunological parameters and dd-cfDNA were assessed using logistic regression analyses. Significant factors identified in the univariate analysis were selected for multivariate analysis if the *P* value of the Wald test was below 0.10. Parameters included in the final multivariate model were identified using a stepwise backward elimination until each parameter was associated with allograft rejection with a *P* value below 0.05. Due to the very limited number of missing values, patients with incomplete variables were excluded (1.4%).

The internal validity of the final model was confirmed using a bootstrap procedure, which involved generating 1,000 datasets derived from resampling the original dataset and permitted the estimation of the bias-corrected 95% CI and the accelerated bootstrap (BCA) OR^30^.

We thereafter investigated whether dd-cfDNA adds value to the standard of care model in detecting rejection. To this aim, we assessed the performances of the model with dd-cfDNA and compared them to those of a model without dd-cfDNA. The performances were assessed with the discrimination ability and calibration. The discrimination ability was evaluated using AUC^31^. Calibration was assessed based on a visual examination of the calibration plots using the predtools package in R. NRI for kidney allograft rejection data was computed using the SurvIDINRI package in R.

A risk score (‘integrative donor-derived cell-free DNA, idd-cfDNA’) to detect kidney allograft rejection was calculated for each patient according to the β-regression coefficients estimated from the final multivariable model, and its discrimination and calibration performances were evaluated in the derivation and in the validation cohorts.

We followed the TRIPOD (Transparent Reporting of a Multivariable Prediction Model for Individual Prognosis or Diagnosis) statement for reporting multivariable prediction model development and validation^32^ and the STARD (Standards for Reporting of Diagnostic Accuracy Studies) checklist for reporting diagnostic accuracy studies^33^.

### Software and package

All analyses were performed using R (v.4.1.2, R Foundation for Statistical Computing), RStudio software (v.1.4.1106) and STATA (v.17). Values of *P* < 0.05 were considered significant and all tests were two-tailed. R packages used were readstata13 (v.0.9.2), ggplot2 (v.3.4.2), GGally (v.2.1.2), cowplot (v.1.1.1), forestmodel (v.0.6.2), ggthemes (v.4.2.4), ggpbur (v.0.6.0), pROC (v.1.18.4), MLmetrics (v.1.1.1), predtools (v.0.0.2), verification (v.1.42), prettyR (v.2.2-3), dplyr (v.1.1.2), magrittr (v.2.0.3), dcurves (v.0.4.0) and SurvIDINRI (v.1.1-2).

### Statistics and reproducibility

No statistical method was used to predetermine sample size, but this study represents the largest study on dd-cfDNA in transplantation. No data were excluded from the analyses and the study was not randomized. The investigators were not blinded to the cfDNA results and outcome assessment.

### Reporting summary

Further information on research design is available in the Nature Portfolio Reporting Summary linked to this article.

## Online content

Any methods, additional references, Nature Portfolio reporting summaries, source data, extended data, supplementary information, acknowledgements, peer review information; details of author contributions and competing interests; and statements of data and code availability are available at 10.1038/s41591-024-03087-3.

### Supplementary information

Supplementary Information Supplementary Tables 1–7 and Figs. 1–4.

Reporting Summary

### Source data

Source Data Fig. 1 Source data of Fig. 1.

Source Data Fig. 2 Source data of Fig. 2.

Source Data Fig. 3 Source data of Fig. 3.

## Figures and Tables

**Extended Data Table 1:**
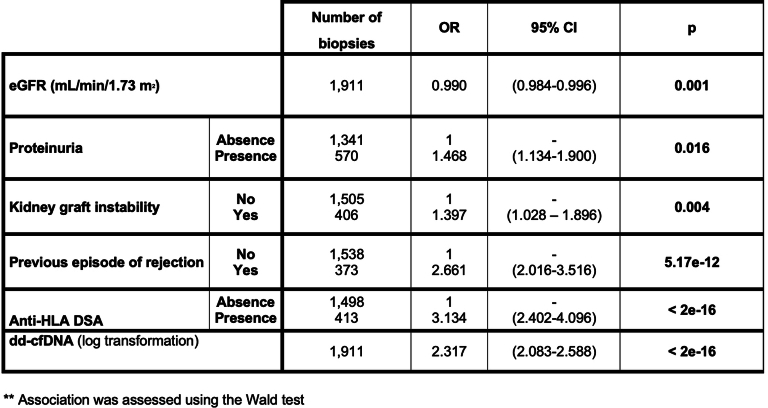
Multivariable analysis of factors associated with rejection in the validation cohort

This table shows the multivariable logistic regression in the validation cohort. Association was assessed using the Wald test.

**Extended Data Table 2:**
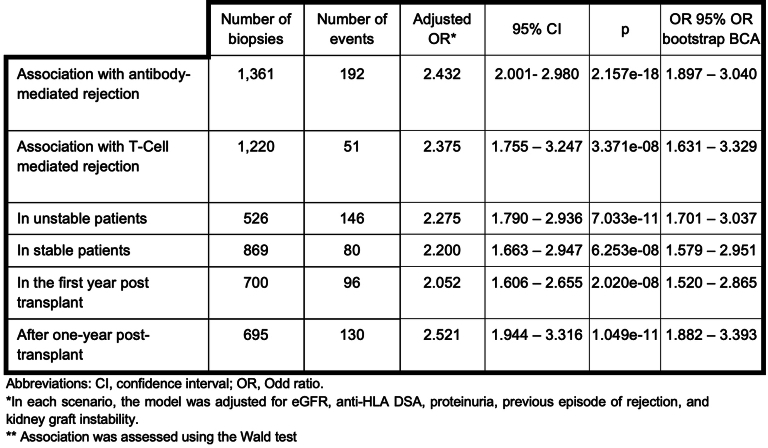
Association of circulating dd-cfDNA with kidney allograft rejection in various clinical scenarios adjusted for eGFR, anti-HLA DSA, proteinuria, previous episode of rejection, and kidney graft instability in the development cohort

In each scenario, the model was adjusted for eGFR, anti-HLA DSA, proteinuria, previous episode of rejection, and kidney graft instability. Association was assessed using the Wald test

**Extended Data Table 3:**
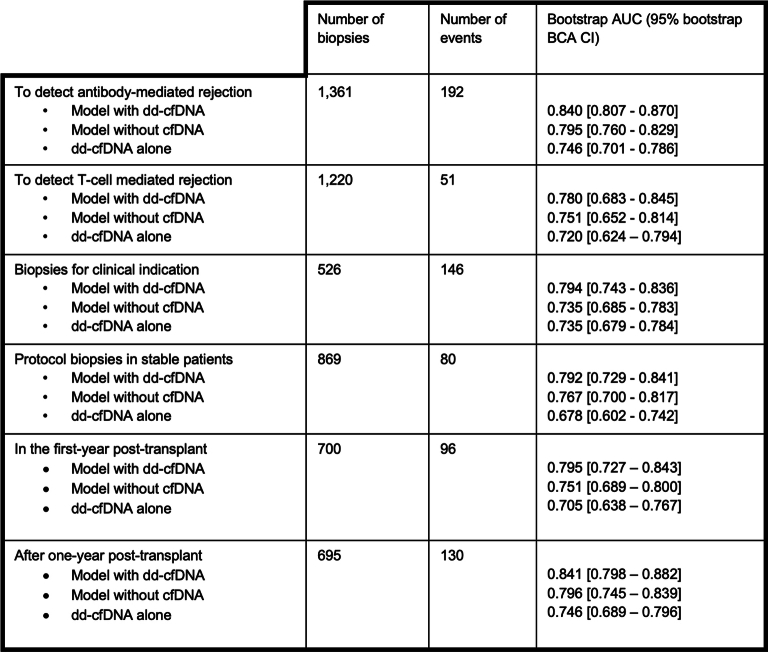
Evaluation of the added value of dd-cfDNA when associated with standard of care parameters (eGFR and proteinuria at time of evaluation, anti-HLA DSA, previous episode of rejection, kidney graft instability), in different clinical scenarios

**Extended Data Table 4:**
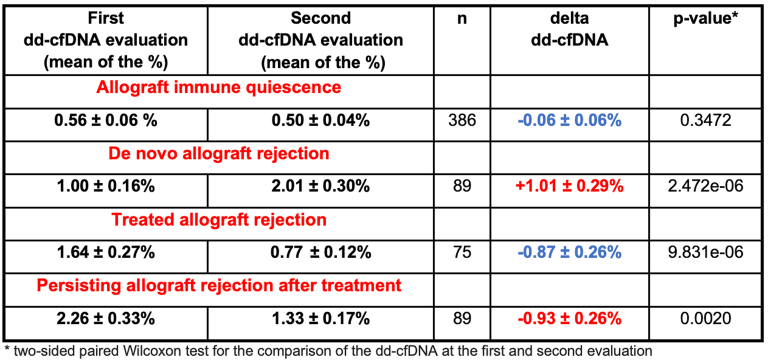
Variation of circulating dd-cfDNA levels according to the 4 prototypical patient scenarios over time

The tests were two-sided paired Wilcoxon tests for the comparison of the dd-cfDNA at the first and second evaluation.

## Data Availability

The raw data collected and generated in the study cannot be made publicly available because of ethical and data protection constraints. Because those data are part of a consortium with other ongoing studies, the data will be shared after review by the consortium and the consent of the different centers. All requests for accessing to the data will be reviewed by representatives from all participating centers involved in this study. If the request is reasonable and complies with both French and the requesting country’s national laws and regulations, anonymized and de-identified data will be shared upon the execution of a data transfer agreement. The data comprise anonymized patient-level clinical data, aggregated clinical data and dd-cfDNA data from the Paris Transplant Institute database and external validation cohort datasets. Timelines vary per request and can take up to a year upon full submission of the request for analysis, decision, anonymization and sharing of the requested data or documents. For all requests, please contact the corresponding author, A.L., directly at alexandreloupy@gmail.com. The protocol and statistical analysis plan have been uploaded to ClinicalTrials.gov. Source data are provided with this paper.

## Code availability

Complete code to reproduce the figures and main analyses is available in the public Synapse database (https://www.synapse.org/#!Synapse:syn58983698/files/53).
