# Supplementary material for: Cell-free DNA for the detection of kidney allograft rejection
Source: Nat Med. 2024 Jun 2;30(8):2320–7. doi: 10.1038/s41591-024-03087-3 (PMC11333280; doi:10.1038/s41591-024-03087-3)

nature medicine

Supplementary information https://doi.org/10.1038/s41591-024-03087-3

Cell-free DNA for the detection of kidney allograft rejection

In the format provided by the authors and unedited

Supplementary Information

1. SUPPLEMENTARY TABLES

Supplementary Table 1: Characteristics at the time of the biopsy with a concomitant dd-cfDNA in the development cohort

Supplementary Table 2: Baseline patient characteristics according to the presence or absence of rejection in the derivation cohort

Supplementary Table 3: Characteristics at the time of the biopsy with a concomitant dd-cfDNA in the external validation cohort

Supplementary Table 4: Baseline patient characteristics according to the presence or absence of rejection in the validation cohort

Supplementary Table 5: Performance metrics of integrative dd-cfDNA in derivation and validation cohorts

Supplementary Table 6: Comparison of the performance metrics (sensitivity, specificity, positive predictive value, negative predictive value, ROC AUC, PRAUC, Brier score) of the model with dd-cfDNA and standard of care parameters, model with only standard of care parameters and dd-cfDNA alone when used as a continuous variable or with different thresholds in the derivation cohort.

Supplementary Table 7: Comparison of the performance metrics (sensitivity, specificity, positive predictive value, negative predictive value, ROC AUC, PRAUC, Brier score) of the model with dd-cfDNA and standard of care parameters, model with only standard of care parameters and dd-cfDNA alone when used as a continuous variable or with different thresholds in the validation cohort.

2. SUPPLEMENTARY FIGURES

Supplementary Figure 1: Association of dd-cfDNA with Chronic Banff scores: IFTA (interstitial fibrosis and tubular atrophy), cv Banff score (arteriosclerosis), ah Banff score (arteriolar hyalinosis), and mm Banff score (mesangial expansion)

Supplementary Figure 2: Distribution of the dd-cfDNA in the validation cohort

Supplementary Figure 3: ROC curve of the integrative dd-cfDNA score in the validation cohort

Supplementary Figure 4: Decision Curve Analysis evaluating the clinical utility of the addition of dd-cfDNA in Predicting rejection compared to standard of care parameters.

1 SUPPLEMENTARY TABLES

Supplementary Table 1: Characteristics at the time of the biopsy with a concomitant dd-cfDNA in the development cohort

	**Derivation cohort (n=1,415)**	
	**N**	
**Estimated GFR,** mean (SD)	1,415	47.65 (19.60)
**Proteinuria (g/g),** mean (SD)	1,411	0.55 (1.39)
**Mean fluorescence intensity of anti-HLA DSAs,** No. (%)	1,400	
**< 500**		861 (61.5)
**500-3000**		426 (30.43)
**3000 – 6000**		54 (3.86)
**> 6000**		59 (4.21)
**Biopsy findings, No.(%)**	1,415	
**Active AMR**		129 (9.12)
**Chronic active AMR**		42 (2.97)
**Inactive AMR**		11 (0.78)
**Equivocal for diagnosis of AMR**		5 (0.35)
**Acute TCMR**		15 (1.06)
**Chronic active TCMR**		19 (1.34)
**Mixed rejection**		17 (1.20)
**Borderline lesions**		19 (1.34)
**Viral nephritis**		20 (1.41)
**Glomerulitis without rejection**		30 (2.12)
**FSGS**		48 (3.39)
**IF-TA**		557 (39.36)
**No specific lesions**		503 (35.55)

Supplementary Table 2: Baseline patient characteristics according to the presence or absence of rejection in the derivation cohort

	No rejection (n=964)		Rejection (n= 170)	
	**N**		**N**	
**Recipient characteristics**				
**Age** (years), mean (SD)	964	52.30 (14.83)	170	51.79 (15.01)
**Sex male**, No. (%)	964	600 (62.24)	170	93 (54.70)
**Cause of end stage renal disease**	964		170	
**Glomerulopathy,** No. (%)		249 (25.83)		45 (26.47)
**Polycystic kidney disease,** No.(%)		161 (16.70)		15 (8.82)
**Interstitial nephritis** (%)		77 (7.99)		17 (10.00)
**Diabetes**, No. (%)		90 (9.34)		14 (8.24)
**Vascular**, No. (%)		77 (7.99)		16 (9.41)
**Other,** No. (%)		122 (12.66)		23 (13.53)
**Unknown etiology**, No (%)		188 (19.50)		40 (23.53)
**Donor characteristics**				
**Age** (years), mean (SD)	960	54.05 (15.91)	169	52.17 (19.65)
**Sex male,** No. (%)	959	501 (52.24)	168	86 (51.19)
**Deceased donor**, No. (%)	963	698 (72.48)	170	122 (71.76)
**Expanded criteria donor,** No. (%)	951	333 (35.02)	169	68 (40.24)
**Transplant baseline characteristics**				
**Prior kidney transplant,** No. (%)	964	141 (14.63)	170	41 (24.12)
**Cold ischemia time** (hours), mean (SD)	953	14.23 (10.37)	167	15.88 (11.31)
**HLA-A/B/DR mismatch**, mean (SD), number	956	3.53 (1.55)	166	3.73 (1.37)
**ABO incompatible transplantation,** No. (%)	960	32 (3.33)	166	2 (1.20)

Abbreviations: SD, Standard deviation; HLA, human leukocyte antigen.

Supplementary Table 3: Characteristics at the time of the biopsy with a concomitant dd-cfDNA in the validation cohort

	Validation cohort (n=2,317)	
**Biopsy findings, No.(%)**	2,317	
**AMR**		352 (15.19)
**TCMR**		224 (9.67)
**Mixed rejection**		103 (4.45)
**Borderline lesions**		183 (7.90)
**Viral nephritis**		100 (4.32)
**Glomerulitis without rejection**		62 (2.68)
**FSGS**		32 (1.38)
**IF-TA**		624 (26.93)
**No specific lesions**		637 (27.49)

Supplementary Table 4: Baseline patient characteristics according to the presence or absence of rejection in the validation cohort

	**No rejection (n=1,252)**		**Rejection (n= 496)**	
	**N**		**N**	
**Recipient characteristics**				
**Age** (years), mean (SD)	1,249	46.36 (18.41)	496	44.64 (17.41)
**Sex male**, No. (%)	1,241	751 (60.52)	494	258 (52.23)
**Cause of end stage renal disease**	1,224		483	
**Glomerulopathy,** No. (%)		361 (29.49)		148 (30.64)
**Polycystic kidney disease,** No.(%)		145 (11.85)		46 (9.52)
**Interstitial nephritis** (%)		145 (11.85)		33 (6.83)
**Diabetes**, No. (%)		203 (16.58)		87 (18.01)
**Vascular**, No. (%)		156 (12.75)		79 (16.36)
**Other,** No. (%)		130 (10.62)		71 (14.70)
**Unknown etiology**, No (%)		84 (6.86)		19 (3.93)
**Donor characteristics**				
**Age** (years), mean (SD)	1,107	44.14 (14.87)	452	39.38 (15.08)
**Sex male,** No. (%)	910	493 (54.18)	330	161 (48.79)
**Deceased donor**, No. (%)	1,250	939 (75.42)	489	352 (71.26)
**Expanded criteria donor,** No. (%)	1,125	191 (16.98)	474	75 (15.82)
**Transplant baseline characteristics**				
**Prior kidney transplant,** No. (%)	1,241	155 (12.49)	491	110 (22.40)
**Cold ischemia time** (hours), mean (SD)	913	13.09 (7.87)	347	12.46 (8.52)
**HLA-A/B/DR mismatch**, mean (SD), number	1,152	3.80 (1.58)	458	4.21 (0.81)
**ABO incompatible transplantation,** No. (%)	1,223	16 (1.31)	481	3 (0.62)

Abbreviations: SD, Standard deviation; HLA, human leukocyte antigen.

Supplementary Table 5: Performance metrics of integrative dd-cfDNA in derivation and validation cohorts

	Cut-off*	Sn	Sp	NPV	PPV	ROC AUC	PRAUC	Brier
Derivation cohort	0.162	0.721	0.784	0.936	0.392	0.821	0.576	0.099
Validation cohort	0.239	0.769	0.768	0.887	0.584	0.838	0.671	0.149

*Cut-off used to optimize the Youden index
Abbreviations: Sn, Sensitivity; Sp Specificity; NPV negative predictive value; PPV positive predictive value; SOC, standard of care

Supplementary Table 6: Comparison of the performance metrics (sensitivity, specificity, positive predictive value, negative predictive value, ROC AUC, PRAUC, Brier score) of the model with dd-cfDNA and standard of care parameters, model with only standard of care parameters and dd-cfDNA alone when used as a continuous variable or with different thresholds in the derivation cohort.

	Cut-off*	Sn	Sp	NPV	PPV	ROC AUC	PRAUC	Brier
Model with dd-cfDNA and SOC parameters	0.162	0.721	0.784	0.936	0.392	0.821	0.576	0.099
Model with only SOC parameters	0.165	0.677	0.780	0.926	0.373	0.777	0.478	0.111
Dd-cfDNA continuous	0.185	0.597	0.796	0.911	0.361	0.730	0.453	0.115
Dd-cfDNA ≥ 0.5 %	0.241	0.540	0.834	0.904	0.386	0.687	0.126	0.123
Dd-cfDNA ≥ 1 %	0.346	0.323	0.953	0.879	0.570	0.638	0.248	0.120

*Cut-off used to optimize the Youden index

Abbreviations: Sn, Sensitivity; Sp Specificity; NPV negative predictive value; PPV positive predictive value; SOC, standard of care

Supplementary Table 7: Comparison of the performance metrics (sensitivity, specificity, positive predictive value, negative predictive value, ROC AUC, PRAUC, Brier score) of the model with dd-cfDNA and standard of care parameters, model with only standard of care parameters and dd-cfDNA alone when used as a continuous variable or with different thresholds in the validation cohort.

	Cut-off*	Sn	Sp	NPV	PPV	ROC AUC	PRAUC	Brier
Model with dd-cfDNA and SOC parameters	0.294	0.762	0.777	0.885	0.591	0.842	0.676	0.145
Model with only SOC parameters	0.340	0.558	0.835	0.817	0.588	0.743	0.575	0.172
Dd-cfDNA continuous	0.300	0.729	0.753	0.868	0.555	0.795	0.586	0.164
Dd-cfDNA ≥ 0.5 %	0.320	0.768	0.695	0.876	0.516	0.732	0.094	0.172
Dd-cfDNA ≥ 1 %	0.383	0.590	0.832	0.828	0.598	0.711	0.184	0.172

*Cut-off used to optimize the Youden index
Abbreviations: Sn, Sensitivity; Sp Specificity; NPV negative predictive value; PPV positive predictive value; SOC, standard of care

2 SUPPLEMENTARY FIGURES

Supplementary Figure 1: Association of dd-cfDNA with Chronic Banff score IFTA (interstitial fibrosis and tubular atrophy), cv Banff score (arteriosclerosis), ah Banff score (arteriolar hyalinosis), and mm Banff score (mesangial expansion)
This figure shows the mean levels of dd-cfDNA according to the chronic Banff score IFTA (interstitial fibrosis and tubular atrophy) (Panel A), cv Banff score (arteriosclerosis) (Panel B), ah Banff score (arteriolar hyalinosis) (Panel C), and mm Banff score (mesangial expansion) (Panel E). Each of these scores ranges from 0 to 3, with higher scores indicating more severe lesions. The lesions were defined according to Banff 2019 classification. Each dot corresponds to an individual dd-cfDNA value. Data are presented as mean values +/- SEM. Comparisons between the groups was performed using two-sided Kruskal-Wallis test with adjustments for multiple comparisons. This figure shows the absence of increment of dd-cfDNA with the severity of the lesions.

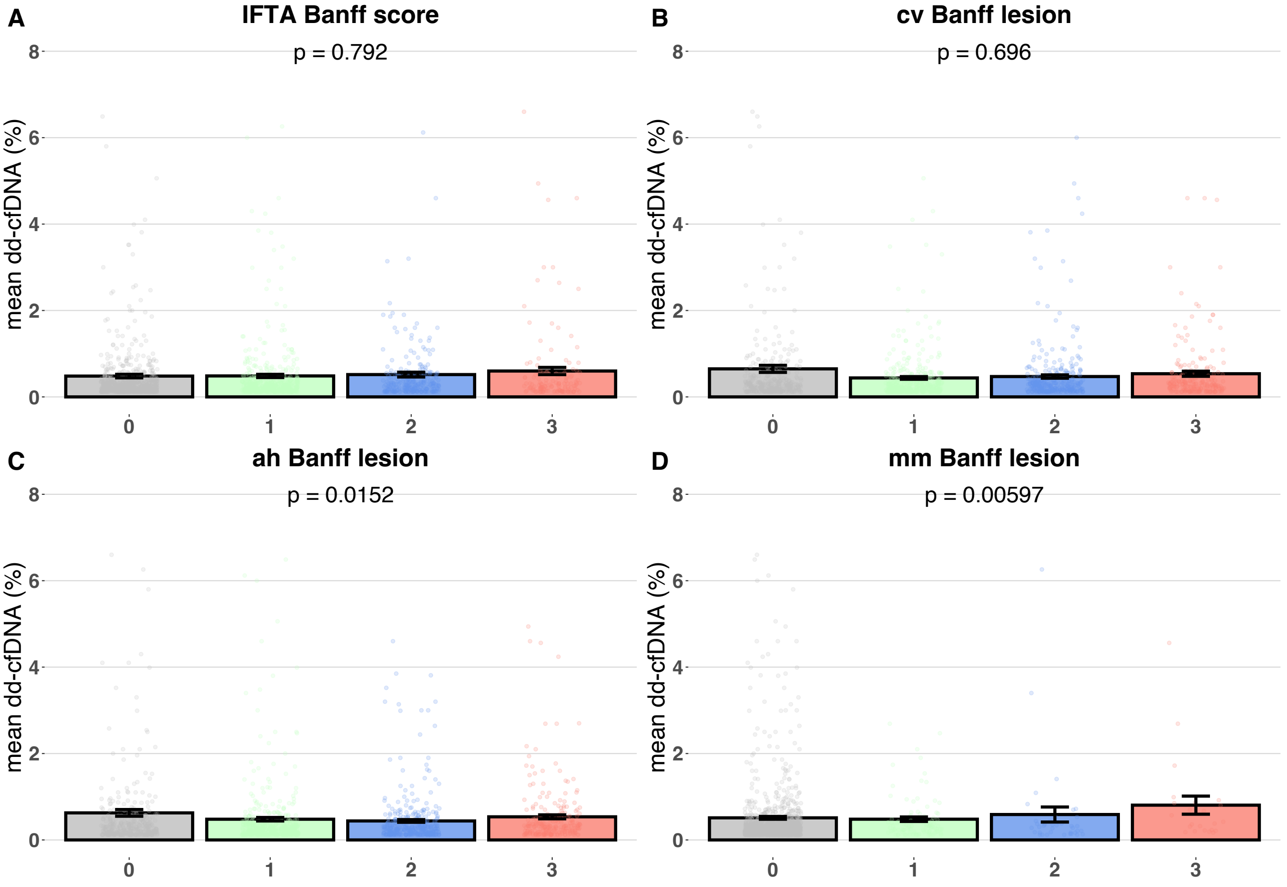

Supplementary Figure 2: Distribution of the dd-cfDNA in the validation cohort
This figure shows the distribution of dd-cfDNA in the validation cohort.

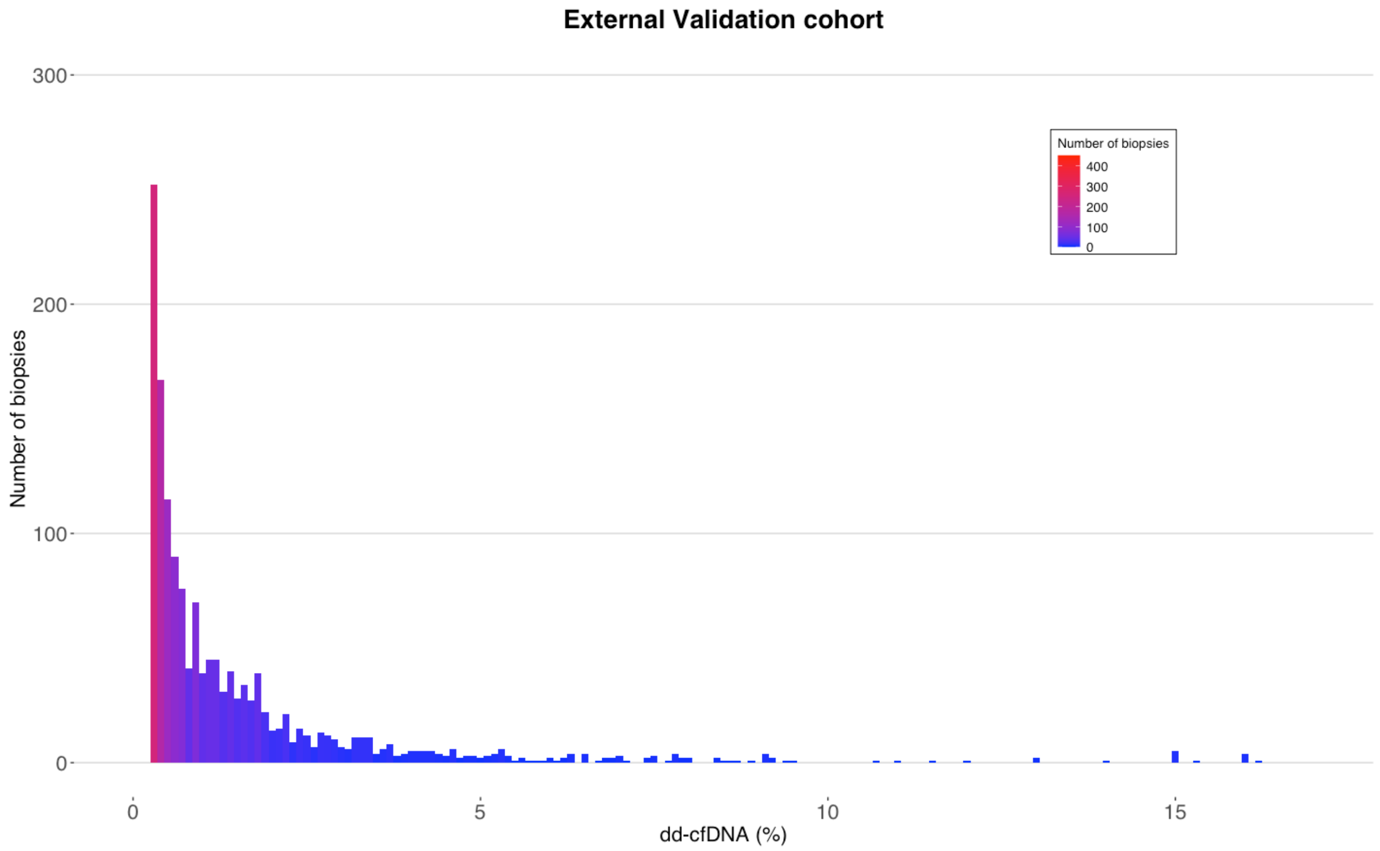

Supplementary Figure 3: ROC curve of the integrative dd-cfDNA score in the validation cohort
This figure shows the receiver-operating (ROC) curve of the integrative dd-cfDNA score in the validation cohort. The area under the curve of the score is equal to 0.838 (biais-corrected 95% CI: 0.817 – 0.855), indicating a good discrimination.

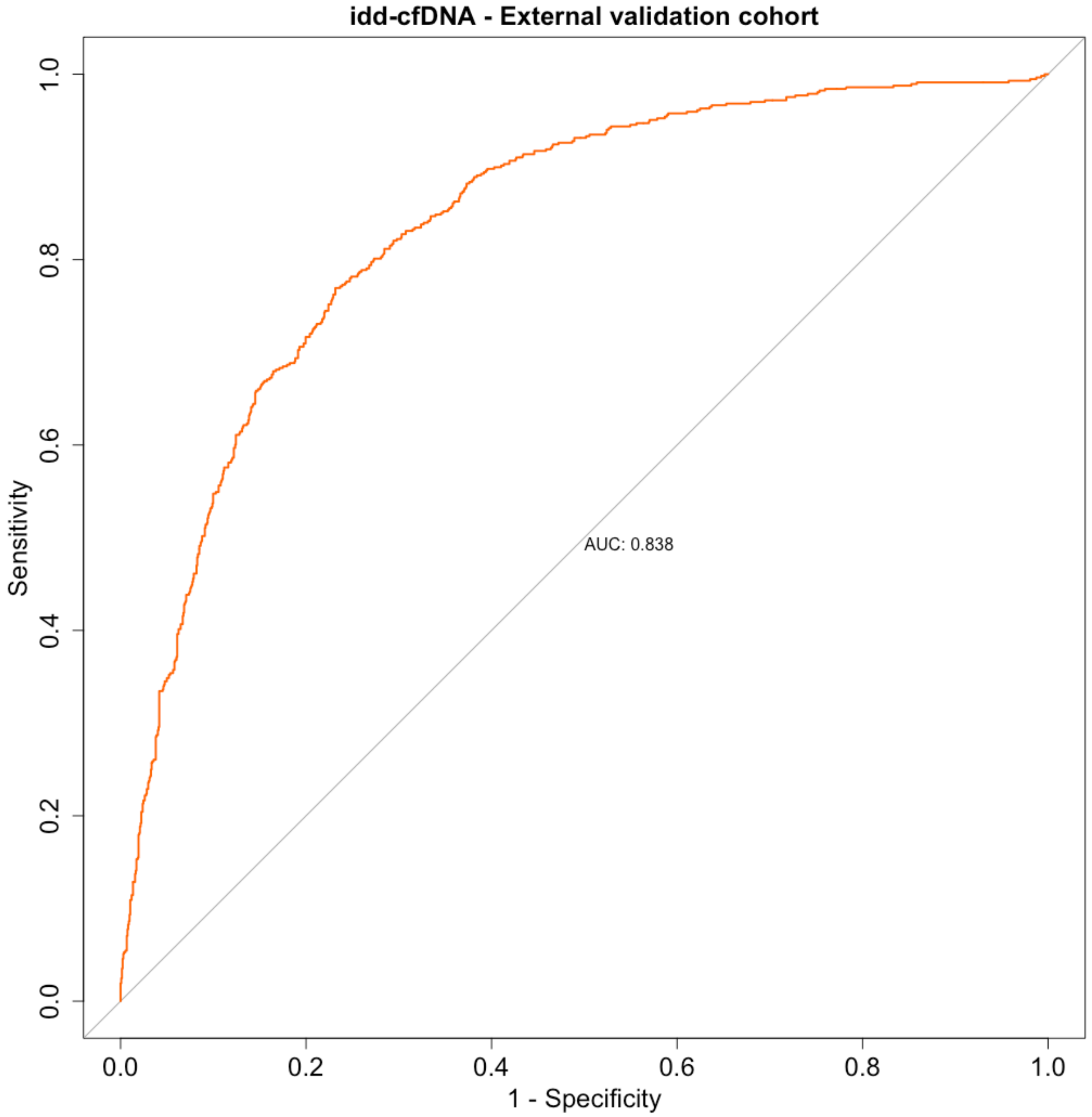

Supplementary Figure 4: Decision Curve Analysis evaluating the clinical utility of the addition of dd-cfDNA in Predicting rejection compared to standard of care parameters.

The vertical axis represents the net benefit, which is a measure of the overall impact of the model on decision-making. The horizontal axis represents the threshold probability, which is the risk level that would prompt a biopsy. The red line corresponds to the assumption that all patients will have a biopsy, the green line that no patients will have a biopsy, the purple line to the decision of performing a biopsy based on the standard of care parameters, and the blue line to the decision of performing a biopsy based on the standard of care parameters with the addition of dd-cfDNA. This figure shows that the net benefit for decisions on the basis of the model integrating dd-cfDNA is higher than that for decisions on the basis the standard of care model.

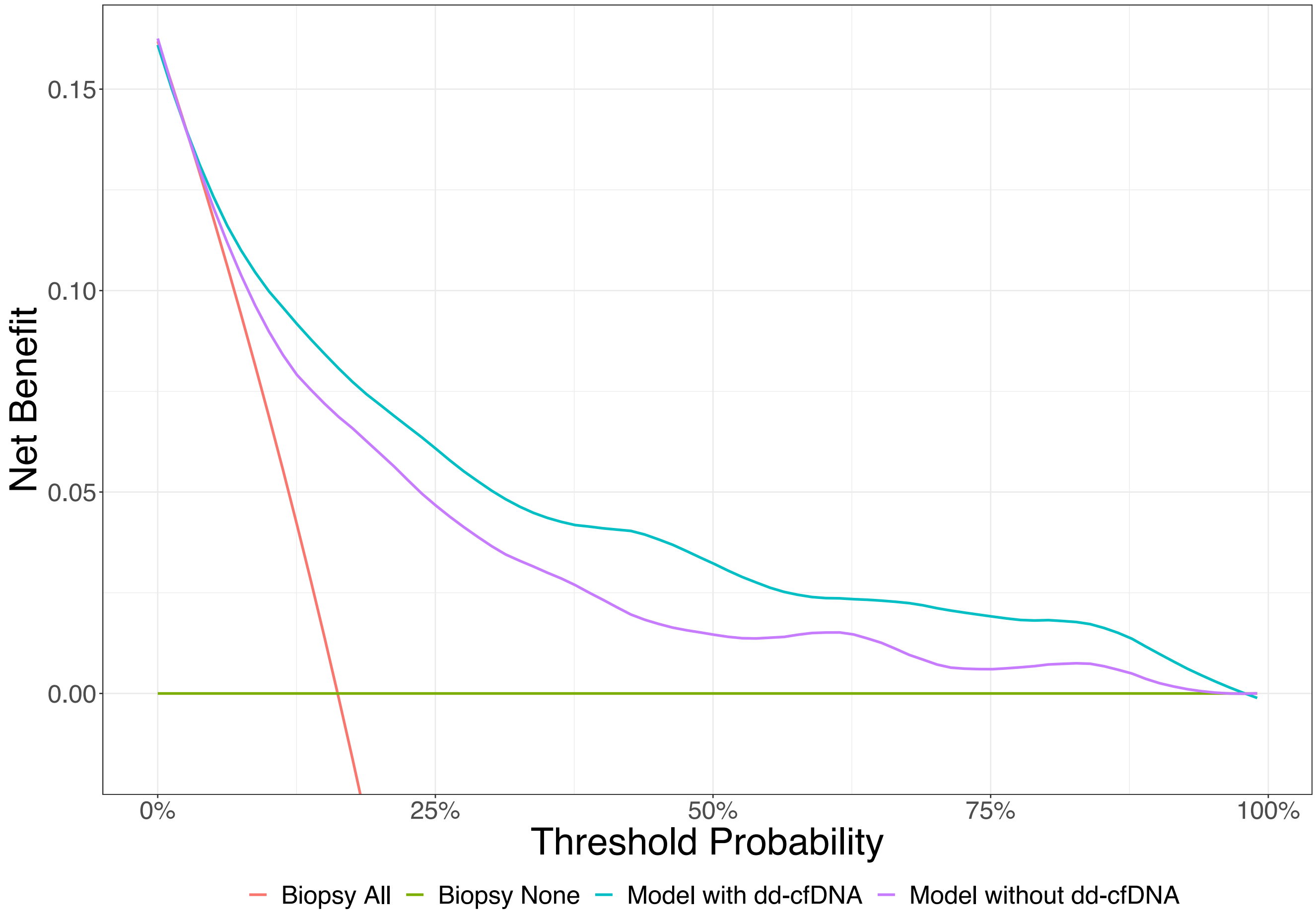

Supplement: Supplementary file 1 — Supplementary Tables 1–7 and Figs. 1–4. [file 41591_2024_3087_MOESM1_ESM.pdf]
