# Supplementary material for: Cell-free DNA for the detection of kidney allograft rejection
Source: Nat Med. 2024 Jun 2;30(8):2320–7. doi: 10.1038/s41591-024-03087-3 (PMC11333280; doi:10.1038/s41591-024-03087-3)
Supplement: Supplementary file 2 — Reporting Summary [file 41591_2024_3087_MOESM2_ESM.pdf]

nature portfolio

Corresponding author(s): Alexandre Loupy (MD, PhD)

Last updated by author(s): May 6, 2024

Reporting Summary

Nature Portfolio wishes to improve the reproducibility of the work that we publish. This form provides structure for consistency and transparency in reporting. For further information on Nature Portfolio policies, see our Editorial Policies and the Editorial Policy Checklist.

Statistics

For all statistical analyses, confirm that the following items are present in the figure legend, table legend, main text, or Methods section.

n/a	Confirmed	
☐	☒	The exact sample size (*n*) for each experimental group/condition, given as a discrete number and unit of measurement
☐	☒	A statement on whether measurements were taken from distinct samples or whether the same sample was measured repeatedly
☐	☒	The statistical test(s) used AND whether they are one- or two-sided<br>*Only common tests should be described solely by name; describe more complex techniques in the Methods section.*
☐	☒	A description of all covariates tested
☒	☐	A description of any assumptions or corrections, such as tests of normality and adjustment for multiple comparisons
☐	☒	A full description of the statistical parameters including central tendency (e.g. means) or other basic estimates (e.g. regression coefficient) AND variation (e.g. standard deviation) or associated estimates of uncertainty (e.g. confidence intervals)
☐	☒	For null hypothesis testing, the test statistic (e.g. *F*, *t*, *r*) with confidence intervals, effect sizes, degrees of freedom and *P* value noted<br>*Give P values as exact values whenever suitable.*
☒	☐	For Bayesian analysis, information on the choice of priors and Markov chain Monte Carlo settings
☒	☐	For hierarchical and complex designs, identification of the appropriate level for tests and full reporting of outcomes
☒	☐	Estimates of effect sizes (e.g. Cohen's *d*, Pearson's *r*), indicating how they were calculated

Our web collection on statistics for biologists contains articles on many of the points above.

Software and code

Policy information about availability of computer code

Data collection	Stata (version 17.0)
Data analysis	Softwares and packages: R (4.1.2), RStudio software (version 1.4.1106), readstata13 (version 0.9.2), ggplot2 (version 3.4.2), GGally (version 2.1.2), cowplot (version 1.1.1), forestmodel (0.6.2), ggthemes (version 4.2.4), ggpbur (version 0.6.0), pROC (version 1.18.4), Mlmetrics (version 1.1.1), predtools (version 0.0.2), verification (version 1.42), prettyR (version 2.2-3), dplyr (version 1.1.2), magrittr (version 2.0.3), dcurves (version 0.4.0), SurvIDINRI (version 1.1-2).

For manuscripts utilizing custom algorithms or software that are central to the research but not yet described in published literature, software must be made available to editors and reviewers. We strongly encourage code deposition in a community repository (e.g. GitHub). See the Nature Portfolio guidelines for submitting code & software for further information.

Data

Policy information about availability of data

All manuscripts must include a data availability statement. This statement should provide the following information, where applicable:
- Accession codes, unique identifiers, or web links for publicly available datasets
- A description of any restrictions on data availability
- For clinical datasets or third party data, please ensure that the statement adheres to our policy

The data collected and generated in the study are not freely available because of ethical and data protection constraints. Because those data are part of a

consortium with other ongoing studies, the data will be shared after review by the consortium and the consent of the different centers. All request for accessing to the data will be reviewed by representatives from all participating centers involved in this study. If the request is reasonable and complies with both French and the requesting country's national laws and regulations, anonymized and de-identified data will be shared upon the execution of a data transfer agreement. The data comprise anonymized patient-level clinical data, aggregated clinical data and dd-cfDNA data from the Paris Transplant Institute database and external validation cohort datasets. Timelines vary per request and can take up to a year upon full submission of the request for analysis, decision, anonymization and sharing of the requested data or documents.. For all requests, please contact the corresponding author, AL, directly at alexandreloupy@gmail.com. The protocol and statistical analysis plan have been uploaded to ClinicalTrials.gov. The multivariable models were developed using standard model libraries from the R software. While the raw data are available from the corresponding author, source data are provided with this paper for main Figures 1, 2, and 3.

Human research participants

Policy information about studies involving human research participants and Sex and Gender in Research.

Reporting on sex and gender

In this research, we included sex variable. Both sex were included and they were self-reported and the number and percentage are reported in the manuscript. In the derivation cohort, 693 (61.11%) were males. There was no missing data. In the validation cohort, 1009 (58.16%) were male and there were missing data for 13 patients (0.74%).

Population characteristics

In the study, we included both adult (over or equal to 18 years age) and paediatric cohorts (under 18 years age). Patients receiving a living or deceased donor kidney allograft were included. Patients with combined organ transplantation, pregnant women, recipients of a graft from a monozygotic twin and patients who had received a bone marrow transplant were excluded. Detailed tables about the population characteristics are available in the manuscript. In the derivation cohort the mean recipient age was 55.2 ± 14.85, with 693 (61.11%) males and in the Validation cohort, the mean recipient age was 45.9 ± 18.14, with 1,009 (58.16%) males.

Recruitment

In each cohort, all patients, or parents/legal guardians if appropriate provided written informed consent to participate.

The derivation cohort consisted of 1,134 patients over 18 years of age prospectively recruited in two French centers (Necker Hospital, Paris and Saint-Louis Hospital, Paris) between April 17, 2013 and June 21, 2021. Patients with combined organ transplantation, pregnant women, recipients of a graft from a monozygotic twin and patients who had received a bone marrow transplant were excluded. All data were anonymised and prospectively entered at the time of transplantation, and were updated at several timepoints (3-, 6- and 12-months post transplantation and then annually), and at each clinical event using a standardised protocol to ensure harmonisation across study centres. Data from the derivation cohort were submitted for an annual audit to ensure data quality.

External validation was conducted on 1,748 kidney transplanted patients from 12 transplantation centers including 322 patients in one Belgium center (Leuven Hospital), and 1,426 patients in eleven North American centers (Cedars-Sinai, Los Angeles, CA [N=284], UCLA, Los Angeles, CA [N=72], University Hospitals, Cleveland, OH [N=30], Emory University, Atlanta, GA [N=136], Intermountain Medical Center Transplant Services, Murray, UT [N=51], Weill-Cornell Medical School and New York Hospital Medical Center, New York, NY [N=34], Virginia Commonwealth University, Richmond, VA [N=327], Washington University School of Medicine in St. Louis, St. Louis MO [N=153], and Transplant Institute, Tampa General Hospital, Tampa, FL [N=132], University of Wisconsin – Madison, Madison, WI [N=207]).

The Belgian and North-American validation cohorts followed the rules applied in each country. In these centres, data sets were collected as part of routine clinical practice and entered in centres' databases in compliance with local and national regulatory requirements, and sent anonymised to the Paris Transplant Group.

The population was unselected. All patients with concomittant dd-cfDNA and a biopsy were included. Because patients without dd-cfDNA were not included, some l diagnoses might be underrepresented in the study.

Ethics oversight

Each patient included in both derivation and validation cohort provided a written informed consent to be included in the French national registry agency (Agence de la Biomédecine) databases CRISTAL and DIVAT. The DIVAT and CRISTAL database networks have been approved by the National French Commission for Bioinformatics, Data, and Patient Liberty: DIVAT: CNIL, registration number: 1016618, validated 8 June 2004; and CRISTAL: CNIL, registration number: 363505, validated 3 April 1996. Informed consent was obtained from the participants at the time of transplantation and from parents or cares for paediatric patients.

Note that full information on the approval of the study protocol must also be provided in the manuscript.

Field-specific reporting

Please select the one below that is the best fit for your research. If you are not sure, read the appropriate sections before making your selection.

☒ Life sciences ☐ Behavioural & social sciences ☐ Ecological, evolutionary & environmental sciences

For a reference copy of the document with all sections, see nature.com/documents/nr-reporting-summary-flat.pdf

Life sciences study design

All studies must disclose on these points even when the disclosure is negative.

Sample size	As the aim of our study was to assess the association of dd-cfDNA with the presence, activity and severity of allograft rejection, and to determine whether dd-cfDNA adds value to standard of care monitoring parameters in detecting kidney allograft rejection, we included as much kidney transplant recipients as possible. We collected multimodal data of 2,882 adult and pediatric patients, totalling 3,732 allograft biopsies, from 14 European and North American transplant referral centers. This represents one of the largest populations of well-phenotyped kidney transplant recipients studied to date. As the observed differences were statistically different, we demonstrated that our sample size was sufficient to answer to the research questions of this work.
Data exclusions	Patients with combined organ transplantation, pregnant women, recipients of a graft from a monozygotic twin and patients who had received a bone marrow transplant were excluded.
Replication	All request for accessing to the data will be reviewed by representatives from all participating centers involved in this study. If the request is reasonable and complies with both French and the requesting country's national laws and regulations, anonymized and de-identified data will be shared upon the execution of a data transfer agreement. For all requests, please contact the corresponding author, AL, directly at alexandreloupy@gmail.com. The protocol and statistical analysis plan have been uploaded to ClinicalTrials.gov.
Randomization	As this is a population based-study, randomization was not applicable. The aim of the study was to assess the association between cf-DNA and the biopsies therefore all the patients had a cf-DNA and a biopsy. The aim of the study was not to compare two groups.
Blinding	Pathologists and physicians were blinded to dd-cfDNA results. CareDx participated in cell free DNA testing and were blinded to clinical informations.

Reporting for specific materials, systems and methods

We require information from authors about some types of materials, experimental systems and methods used in many studies. Here, indicate whether each material, system or method listed is relevant to your study. If you are not sure if a list item applies to your research, read the appropriate section before selecting a response.

Materials & experimental systems

n/a	Involved in the study	
☒	☐	Antibodies
☒	☐	Eukaryotic cell lines
☒	☐	Palaeontology and archaeology
☒	☐	Animals and other organisms
☐	☒	Clinical data
☒	☐	Dual use research of concern

Methods

n/a	Involved in the study	
☒	☐	ChIP-seq
☒	☐	Flow cytometry
☒	☐	MRI-based neuroimaging

Clinical data

Policy information about clinical studies

All manuscripts should comply with the ICMJE guidelines for publication of clinical research and a completed CONSORT checklist must be included with all submissions.

Clinical trial registration	NCT05995379
Study protocol	Full trial protocol can be accessed at ClinicalTrials.gov: NCT05995379
Data collection	In each cohort, all patients, or parents/legal guardians if appropriate provided written informed consent to participate.<br><br>The derivation cohort consisted of 1,134 patients over 18 years of age prospectively recruited in two French centers (Necker Hospital, Paris and Saint-Louis Hospital, Paris) between April 17, 2013 and June 21, 2021. Patients with combined organ transplantation, pregnant women, recipients of a graft from a monozygotic twin and patients who had received a bone marrow transplant were excluded. All data were anonymised and prospectively entered at the time of transplantation, and were updated at several timepoints (3-, 6- and 12-months post transplantation and then annually), and at each clinical event using a standardised protocol to ensure harmonisation across study centres. Data from the derivation cohort were submitted for an annual audit to ensure data quality. All data from Paris-Necker and Paris-Saint Louis hospitals were extracted from the prospective Paris Transplant Group Cohort data cohort. CNIL, Registration number: 363505, validated on the 8th of June 2004. The database networks have been approved by the National French Commission for bioinformatics data and patient liberty and codes were used to ensure strict donor and recipient anonymity and blind access<br><br>External validation was conducted on 1,748 kidney transplanted patients from 12 transplantation centers including 322 patients in one Belgium center (Leuven Hospital), and 1,426 patients in eleven North American centers (Cedars-Sinai, Los Angeles, CA [N=284], UCLA, Los Angeles, CA [N=72], University Hospitals, Cleveland, OH [N=30], Emory University, Atlanta, GA [N=136], Intermountain

Medical Center Transplant Services, Murray, UT [N=51], Weill-Cornell Medical School and New York Hospital Medical Center, New York, NY [N=34], Virginia Commonwealth University, Richmond, VA [N=327], Washington University School of Medicine in St. Louis, St. Louis MO [N=153], and Transplant Institute, Tampa General Hospital, Tampa, FL [N=132], University of Wisconsin – Madison, Madison, WI [N=207]).

The Belgian and North-American validation cohorts followed the rules applied in each country. In these centres, data sets were collected as part of routine clinical practice and entered in centres' databases in compliance with local and national regulatory requirements, and sent anonymised to the Paris Transplant Group.

Outcomes

The outcome of interest was biopsy-proven rejection comprising antibody mediated rejection, T-cell mediated rejection and mixed rejection. Allograft rejection were detected in screening allograft biopsies performed at 3 months and 1 year after transplant and/or in for cause biopsies performed at any time post transplantation in unstable patients. All allograft biopsies were assessed according to the Banff 2019 classification.

In the derivation cohort, allograft biopsies were scored and graded from 0 to 3 according to the updated Banff criteria for allograft pathology for the following histological factors: glomerular inflammation (glomerulitis), tubular inflammation (tubulitis), interstitial inflammation, endarteritis, peritubular capillary inflammation (capillaritis), transplant glomerulopathy, interstitial fibrosis, tubular atrophy, arteriolar hyalinosis and arteriosclerosis. Additional diagnoses provided by the biopsy (e.g., the diagnoses of primary disease recurrence, BK virus nephropathy) were recorded. The biopsy sections (4 µm) were stained with periodic acid-Schiff, Masson's trichrome, and hematoxylin and eosin. C4d staining was performed via immunohistochemical analysis on paraffin sections using polyclonal human anti-C4d antibodies. Also in the validation cohorts, the Banff criteria for the individual histological lesions were assessed in each biopsy included in the study.
